# Recent progress on the total synthesis of acetogenins from Annonaceae

**DOI:** 10.3762/bjoc.4.48

**Published:** 2008-12-05

**Authors:** Nianguang Li, Zhihao Shi, Yuping Tang, Jianwei Chen, Xiang Li

**Affiliations:** 1 Department of Medicinal Chemistry, Nanjing University of Chinese Medicine, No. 138, Xianlindadao, Nanjing, Jiangsu 210046, P. R. China. Tel & Fax: +86-25-85811512; 2 Division of Organic Chemistry, China Pharmaceutical University, Nanjing, Jiangsu 211198, P. R. China; 3 Jiangsu Key Laboratory for TCM Formulae Research, Nanjing University of Chinese Medicine, Nanjing, Jiangsu 210046, P. R. China.

**Keywords:** annonaceous acetogenins, antitumor, natural product, total synthesis

## Abstract

An overview of recent progress on the total synthesis of acetogenins from Annonaceae during the past 12 years is provided. These include mono-tetrahydrofurans, adjacent bis-tetrahydrofurans, nonadjacent bis-tetrahydrofurans, tri-tetrahydrofurans, adjacent tetrahydrofuran-tetrahydropyrans, nonadjacent tetrahydrofuran-tetrahydropyrans, mono-tetrahydropyrans, and acetogenins containing only γ-lactone. This review emphasizes only the first total synthesis of molecules of contemporary interest and syntheses that have helped to correct structures. In addition, some significant results on the novel synthesis and structure–activity relationship studies of annonaceous acetogenins are also introduced.

## Introduction

Annonaceous acetogenins (ACGs) constitute a series of natural products isolated exclusively from Annonaceae species [1–5] that are widely distributed in tropical and sub-tropical regions. Since uvaricin [6], the first acetogenin identified from the roots of *Uvaria acuminata*, more than 400 members of this family of compounds have been isolated from 51 different species [7].

The common skeleton is most often characterised by an unbranched C_32_ or C_34_ fatty acid ending in a γ-lactone. Several oxygenated functions, such as hydroxyls, ketones, epoxides, tetrahydrofurans (THF) and tetrahydropyrans (THP), may be present, as well as double and triple bonds. Thus several types of ACGs have been characterised, based on the nature of the functional groups which are present. These including mono-THF, adjacent bis-THF, nonadjacent bis-THF, tri-THF, adjacent tetrahydrofuran-tetrahydropyran (THF-THP), nonadjacent THF-THP, mono-THP, and acetogenins containing only γ-lactones.

These compounds are known to exhibit a broad range of biological activities, the precedent for which came from early South American populations, who used extracts of Annonaceae plants as pesticidal and antiparasitic agents [8]. The proven activities of the acetogenins now include (but are not limited to): pesticidal, antifeedant, antiprotozoal, immunosuppressive and probably most importantly, antitumor [3]. In this respect they are known to be very potent cytotoxic compounds, targeting the reduced nicotinamide adenine dinucleotide (NADH): ubiquinone oxidoreductase (also known as complex I) which is a membrane bound protein of the mitochondrial electron transport system, and the ubiquinone linked NADH oxidase in the plasma membrane of cancerous cells [9–10]. Inhibition by these mechanisms results in adenosine triphosphate (ATP) deprivation, which leads to apoptosis of the highly energy demanding tumor cells [11]. The acetogenins are now considered as the most potent (effective in nanomolar concentrations) known inhibitors of the mitochondrial complex I [9,12]. More recently the annonaceous acetogenins have also been shown to overcome resistance in multidrug resistant (MDR) tumors [13].

Because of their structural diversity and the numerous biological properties, many authors are working on the total synthesis of ACGs. Previous reviews on the total synthesis of ACGs have already been published [14–18]; since 1996 over 100 total syntheses of all types of ACGs have appeared in the literature, illustrating the creativity of chemists. Convergent, linear, and biomimetic approaches have been used, relying on the use of cheap chiral starting materials (*e.g.* amino acids, sugars, tartaric acid, *etc.*) or on asymmetric reactions {*e.g.* Sharpless asymmetric epoxidation (AE), Sharpless asymmetric dihydroxylation (AD), diastereoselective Williamson etherification, *etc.*}. Semi-synthesis of natural ACGs as well as derivatised ACGs (*e.g.* amines, esters, and glycosylated ACGs) and preparation of structural analogues (*e.g.* simplified mimics, chimeras) have also been reported. This overview covers only examples of the first total synthesis and the syntheses that helped to correct structures achieved during the past 10 years (largely up to 2007). Indeed, total synthesis is a key tool for the complete structure determination of ACGs, since several absolute configurations of stereogenic centres are rather difficult to determine without comparison of the spectroscopic data, and/or the chromatographic properties of several stereoisomers. This review of total synthesis of every type of ACGs is presented in order of publication.

## Review

### Total synthesis of ACGs

The significant biological activity of the acetogenins, as well as their interesting and diverse structures, has stimulated substantial interest in their chemical synthesis.

### 1 Mono-THF ACGs

#### Total synthesis of longifolicin

**Scheme 1:**
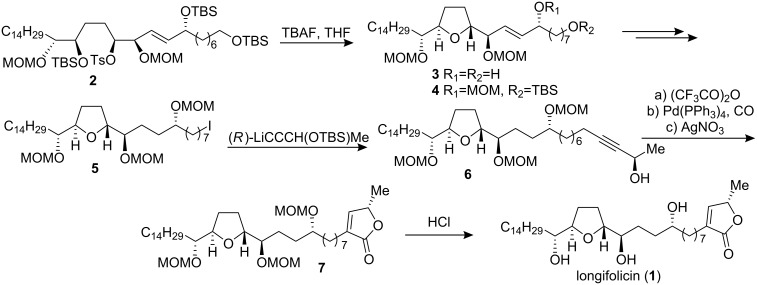
Total synthesis of longifolicin by Marshall’s group.

In 1998, Marshall’s group [19] reported the total synthesis of longifolicin (**1**) (Scheme 1). Treatment of the advanced intermediate **2** with tetrabutylammonium fluoride (TBAF) in THF gave the THF product **3**. Attachment of the butenolide moiety to the THF intermediate **4** was achieved through introduction of a chiral propargylic alcohol segment to yield **6**, to which an allenyl Pd hydrocarbonylation methodology was applied to form the butenolide **7**. The MOM protecting groups in **7** were cleaved to give longifolicin (**1**) in high yield. The ^1^H and ^13^C NMR spectra were identical to those of an authentic sample. Furthermore, the rotation ([α]_D_ +13.5, *c* 0.37, CH_2_Cl_2_) and mp (79–80 °C) were in close agreement with the reported values ([α]_D_ +13.0, *c* 0.001, CH_2_Cl_2_; mp 83 °C) [20]; so this total synthesis confirmed the structural assignment of longifolicin.

#### Total synthesis of corossoline

**Scheme 2:**
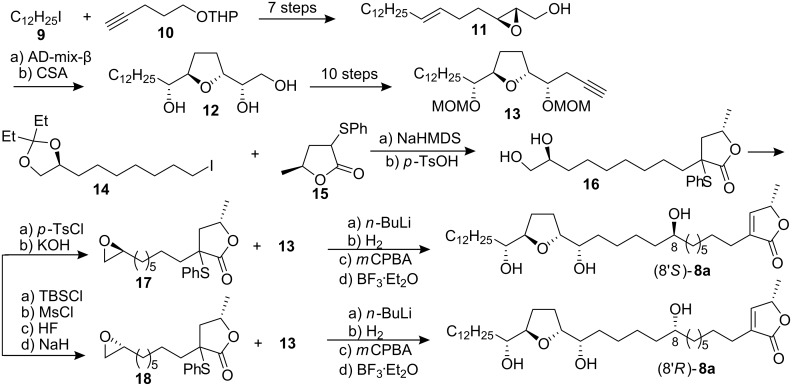
Total synthesis of corossoline by Tanaka’s group.

Corossoline (**8**) was originally isolated [21] from the seeds of *Annona muricata* in 1991. Its absolute stereochemistry except for the C-8′ position was deduced by applying Mosher’s new methodology to the monotetrahydrofuranyl ACGs such as reticulatacin [22] and by a total synthesis of (8′*RS*)-corossoline by Wu’s group [23–24]. In 1996, Tanaka’s group [25] reported the total synthesis of two possible diastereomers (8′*R*)- and (8′*S*)-**8a** to confirm the stereochemistry of the C-8′ hydroxyl group (Scheme 2). Their synthetic approach started from 1-iodododecane (**9**) and 5-(tetrahydropyran-2-yloxy)pent-1-yne (**10**). Asymmetric dihydroxylation with AD-mix-β on **11** and subsequent acid-catalyzed cyclization with camphorsulfonic acid (CSA) resulted in THF ring-containing building block **12**, which was converted into alkyne **13**. The alkylation of iodide **14** with the sodium enolate of **15** afforded **16**. Transformation of **16** following two different procedures afforded isomeric epoxides **17** and **18**, respectively. Coupling between the lithium salt of **13** and **17** (or **18**) followed by hydrogenation, oxidation and deprotection afforded (8′*S*)- or (8′*R*)-**8a**. Comparison of the mp, [α]_D_, IR and NMR data of both synthetic materials with those reported for natural corossoline did not allow for the strict determination of the configuration at the C-8′ hydroxyl group of **8**.

**Scheme 3:**
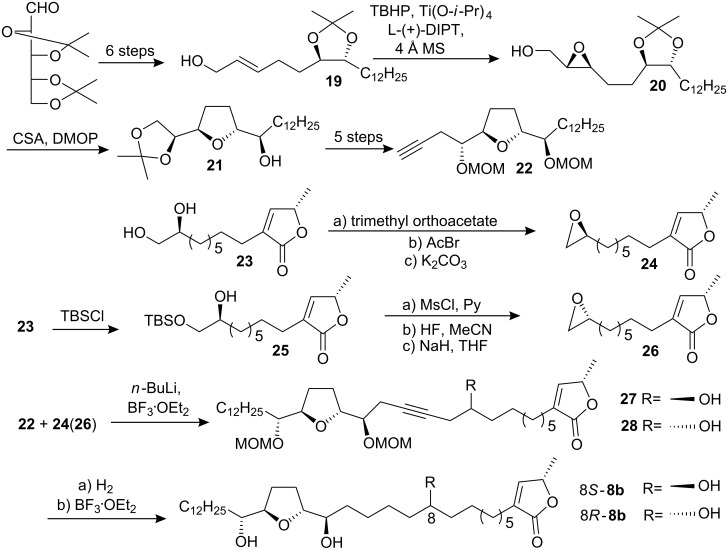
Total synthesis of corossoline by Wu’s group.

To establish the previously undefined configuration at C-8 of the natural **8**, in 1999, Wu’s group [26] reported the synthesis of (8*R*)- and (8*S*)-corossoline and assigned the absolute configuration of natural-**8** at C-8 to *R* (Scheme 3). In their synthesis, asymmetric epoxidation of **19** directed by L-(+)-diisopropyl tartrate gave epoxy alcohol **20**; the THF ring of **21** was then constructed under CSA catalyzed one-pot transformation. After deprotection of the isopropylidene acetal of **21** and oxidative cleavage of the resulting diol, the resultant aldehyde was treated with chiral allenylboronic ester to afford **22**. The terminal epoxides **24** and **26** were prepared from the same intermediate **23**. Coupling of **22** with **24** (or **26**) in the same manner followed by hydrogenation and removal of the MOM protecting group gave (8*S*)-**8b** (or (8*R*)-**8b**) respectively. Comparison of the physical data of both synthetic corossolines with those reported for the natural one showed that the configuration at C-8 of the natural corossoline was highly likely to be *R*.

#### Synthesis of pseudoannonacin A

**Scheme 4:**
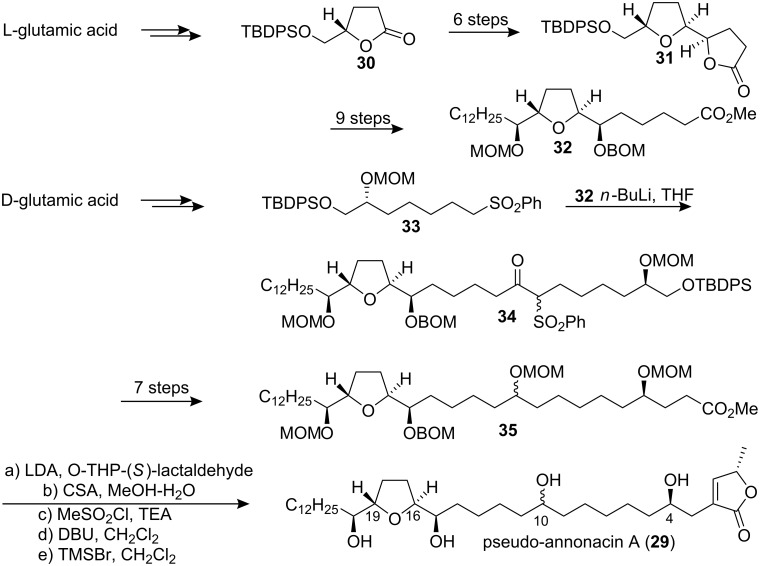
Total synthesis of pseudo-annonacin A by Hanessian’s group.

Annonacin A was isolated from seeds of *Annona squamosa* and obtained as an amorphous solid, [α]_D_ +23.8 (*c* 0.4, CH_2_Cl_2_) [27]. It was characterized spectroscopically, and the relative configuration of the central THF ring was assigned as being *threo-trans-erythro *(C_16_*_R_*,C_19_*_R_*). But the configurations at C-4 and C-10 remained unknown. In 1998, Hanessian’s group [28] reported the stereocontrolled first total synthesis of a diastereomer of the presumed mono-THF type acetogenin annonacin A utilizing the “Chiron approach” (Scheme 4). The well-known lactone **30** obtained from L-glutamic acid was elaborated to a lactone intermediate **31**; further manipulation and chain elongation at both ends of **31** produced an advanced intermediate **32**. The anion of the phenyl sulfone **33**, readily prepared from D-glutamic acid, was condensed directly with the ester function of **32** to give the α-keto sulfone **34**, which was futher elaborated to ester **35**. Condensation of the enolate derived from **35** with O-THP (*S*)-lactaldehyde followed by mesylation, elimination and deprotection afforded **29**. The synthetic product (a mixture of epimers at C-10) had spectroscopic data identical to those of the natural product, but a different optical rotation. They designated the (C_15_*_R_*,C_16_*_S_*,C_19_*_S_*,C_20_*_S_*) *erythro-trans-threo* isomer **29** as pseudo-annonacin A. The actual configuration of the natural product remains unknown.

#### Total synthesis of tonkinecin

**Scheme 5:**
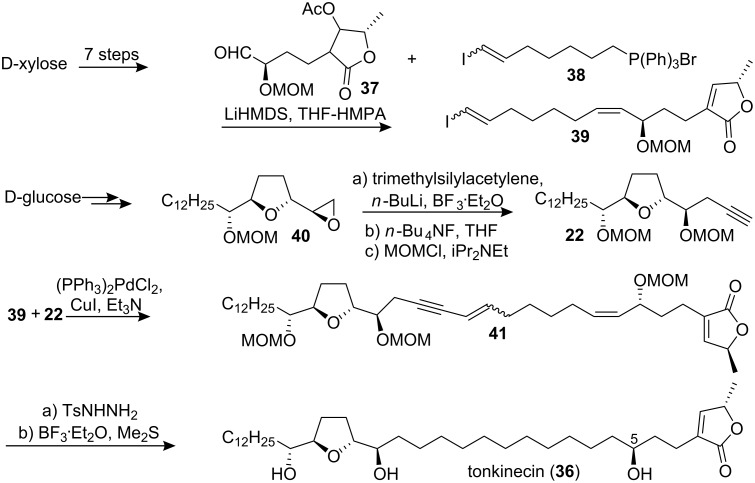
Total synthesis of tonkinecin by Wu’s group.

Tonkinecin (**36**) is a mono-THF acetogenin with a hydroxyl group at C-5, and was recently isolated from roots of *Uvaria tonkinesis* by Yu’s group [29]. This compound has demonstrated potent cytotoxicity against hepatoma (Bel 7402) (IC_50_ = 1.5 µM), gastrocarcinoma (BGC) (IC_50_ = 5.1 µM), colon adenocarcinoma (HCT-8) (IC_50_ = 0.38 µM), and leukemia (HL-60) (IC_50_ = 0.52 µM) human tumor cell lines [29]. Tonkinecin (**36**) was firstly synthesized by Wu’s group in 1999 [30] which used a palladium-catalyzed cross-coupling reaction between the butenolide **39** and the THF unit **22** as the key step for constructing the backbone of **36** (Scheme 5). The synthesis of aldehyde **37** began with D-xylose and involved construction of a γ-lactone moiety utilizing Wu’s own methodology. Wittig reaction of the aldehyde **37** and the Wittig reagent **38** furnished the butenolide unit **39**. The tetrahydrofuran part of **22** was constructed from D-glucose *via* epoxide **40**. The entire carbon skeleton of **41** was constructed by Pd(0)-catalyzed cross-coupling reaction between **39** and **22**. Selective hydrogenation and removal of the MOM protecting group gave tonkinecin (**36**). The physical data of their synthetic sample were identical to those of the natural one. In 2001, the full details of this total synthesis were reported [31].

#### Total synthesis of gigantetrocin A

**Scheme 6:**
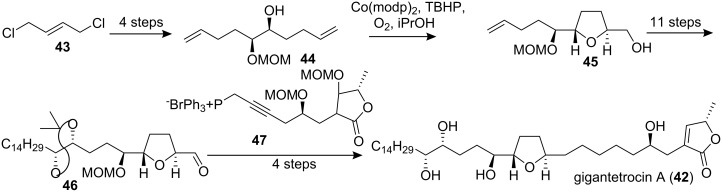
Total synthesis of gigantetrocin A by Shi’s group.

Gigantetrocin A (**42**) was isolated by McLaughlin’s group from *Goniothalamus giganteus* [32] and showed significant and selective cytotoxicity to human tumor cells in culture [32–33]. In 2000, Shi’s group reported the first simple total synthesis of gigantetrocin A (Scheme 6) [34]. They obtained the *trans*-THF ring building block **45** by means of the Sharpless AD reaction and cyclization catalyzed by Co(modp)_2_ (the Mukaiyama epoxidation method) under mild reaction conditions.

The connection of the THF unit **46** with the γ-lactone segment **47** was carried out by means of a Wittig reaction. The target compound **42** had specific rotation and spectral data matching those reported in the literature [32].

#### Total synthesis of annonacin

**Scheme 7:**
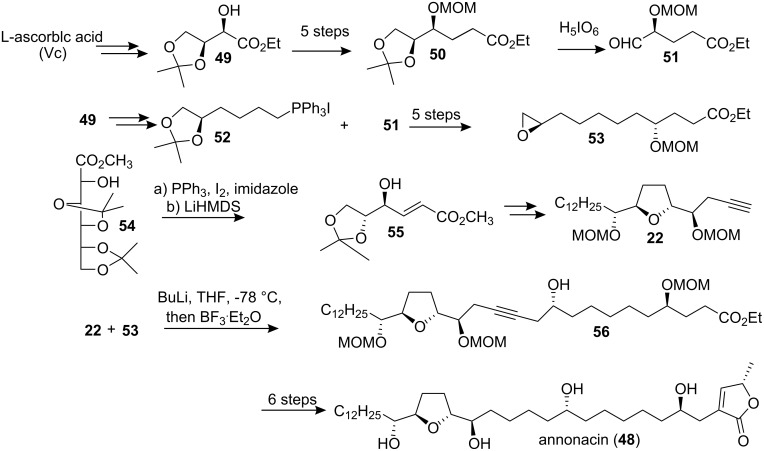
Total synthesis of annonacin by Wu’s group.

Annonacin (**48**), the first mono-THF acetogenin discovered, was isolated by Cassady’s group from the stembark of *Annona densicoma* in 1987 [35]. This compound demonstrated astrocytoma reversal (9ASK) activity (15−30% reversal at 100 µg/mL) and high cytotoxicity against human nasopharyngeal carcinoma (KB) and mouse leukemia (P388) [35–36]. In 2000, Wu’s group reported the first total synthesis of annonacin (Scheme 7) [37]. In their synthetic route, the *R*-hydroxy ester **49** obtained from L-ascorbic acid was elongated by two carbons to give ester **50** using a four-step sequence, the protected diol of which was then treated with H_5_IO_6_ to give the chiral aldehyde **51**. On the other hand, **49** was converted to phosphonium salt **52**, which was treated with aldehyde **51**, and the resulting alkene was further elaborated to afford the epoxide **53**. Next, the lithiated derivative of THF alkyne **22**, which was prepared from the D-glucono-*δ*-lactone-derived α-hydroxyl ester **54** through Sharpless AD reaction as a key step, was treated with epoxide **53** in the presence of BF_3_·Et_2_O to afford alkynol **56**. Catalytic hydrogenation of **56** and subsequent construction of the butenolide segment finished the total synthesis of annonacin (**48**), whose *R_f_* value and spectroscopic data were identical to those reported for the natural product. In 2001, the full details of this total synthesis were reported [31].

#### Total synthesis of solamin

**Scheme 8:**
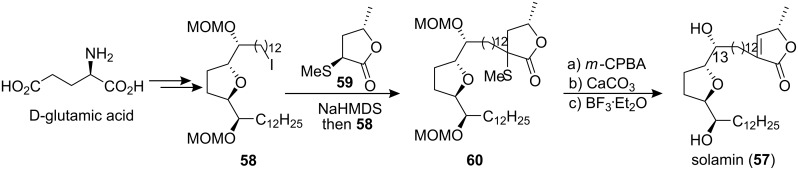
Total synthesis of solamin by Kitahara’s group.

Solamin (**57**), a cytotoxic mono-tetrahydrofuranic γ-lactone acetogenin isolated from *Annona muricata* seeds in 1991 [38], was synthesized by Kitahara’s group in 1999 [39] through a direct coupling reaction (Scheme 8). The mono-THF unit **58**, which was prepared from D-glutamic acid, was treated with the sodium enolate of **59** to afford the main structure **60**. Oxidation of the sulfide **60** followed by thermal elimination and deprotection completed the total synthesis of solamin (**57**). The data of the synthetic **57** were identical to those of an authentic sample [38].

**Scheme 9:**
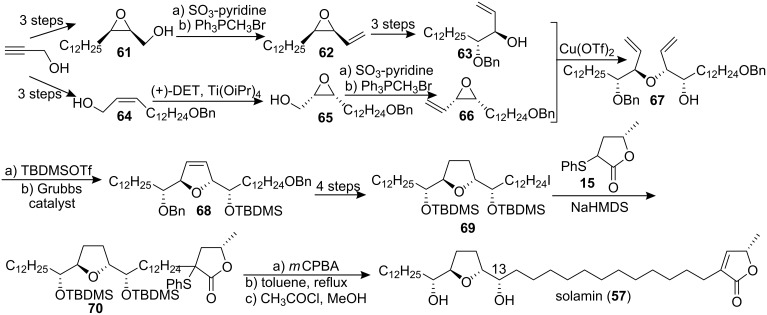
Total synthesis of solamin by Mioskowski’s group.

In their total synthesis of solamin (**57**), Mioskowski’s group [40] reported the first application of the RCM reaction using a ruthenium catalyst thus demonstrating the efficiency of this reaction for the construction of ACGs (Scheme 9). Both allyl alcohol **63** and vinyl-substituted epoxide **66** were synthesized via alkyne reduction yielding (*E*)- or (*Z*)-allylic alcohol followed by Sharpless AE using (+)- or (−)-DET. This synthesis was quite flexible and all stereoisomers of the central THF core of solamin were easily obtained. The allyl alcohol **63** and the vinyl epoxide **66** were then coupled to construct the solamin skeleton **67**. Subjecting of **67** to 1,3-dimesitylimidazol-2-ylidene ruthenium benzylidene catalyst (RuCl_2_(=C(H)-Ph)(PCy_3_)(IMes)) after protection of the free hydroxyl group afforded the RCM product **68**. Hydrogenation of the double bond of dihydrofuran **68** and iodination of the primary alcohol gave **69**, which was then utilized to alkylate the sodium enolate of lactone **15** to afford **70**. Oxidation of the sulfide **70** followed by thermal elimination and finally removal of the two silyl protective groups gave solamin (**57**). But after careful comparison the configuration of the OH-group in C13 between the literature for isolation [38] and this total synthesis by Mioskowski’s group, we found that Mioskowski’s group may have made a mistake in the configuration of the OH-group in C13, and the configuration of the OH-group in C13 should be *R* as in Scheme 8, not *S* in Scheme 9.

#### Total synthesis of *cis*-solamin

**Scheme 10:**
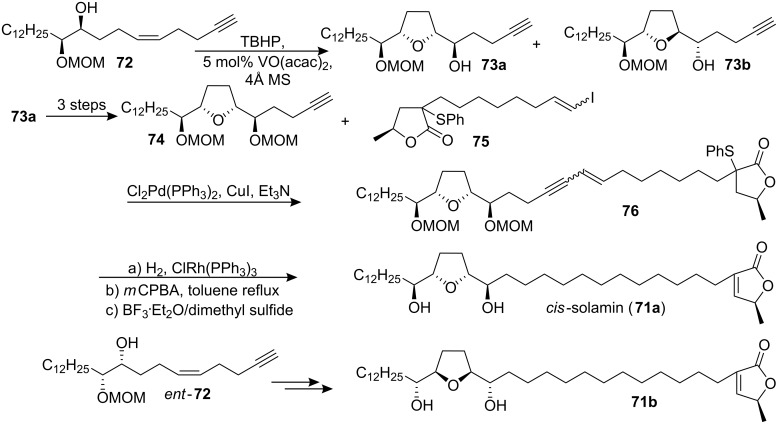
Total synthesis of *cis*-solamin by Makabe’s group.

*cis*-Solamin is a mono-THF acetogenin isolated from *Annona muricata* in 1998 [41]. To establish the absolute configuration of *cis*-solamin, two candidates **71a** and **71b** were synthesized by Makabe’s group in 2002 employing a TBHP-VO(acac)_2_ diastereoselective epoxidation followed by a cyclization strategy (Scheme 10) [42]. In their synthesis, diastereoselective epoxidation of **72** and spontaneous cyclization afforded the diastereomers **73a** and **73b**. Protection of the hydroxyl group of **73a** as a MOM ether afforded **74**, which was coupled with the γ-lactone precursor **75** by a Sonogashira cross-coupling reaction to give compound **76**. Catalytic hydrogenation of **76** using Wilkinson’s catalyst and oxidation of the sulfur with *m*CPBA followed by thermal elimination afforded the candidate **71a**. The other candidate **71b** was synthesized using the same reaction sequence as that employed for **71a**. By comparison of the optical rotation of the two possible diastereomers, it was suggested that the absolute configuration of natural *cis*-solamin was **71a**.

**Scheme 11:**
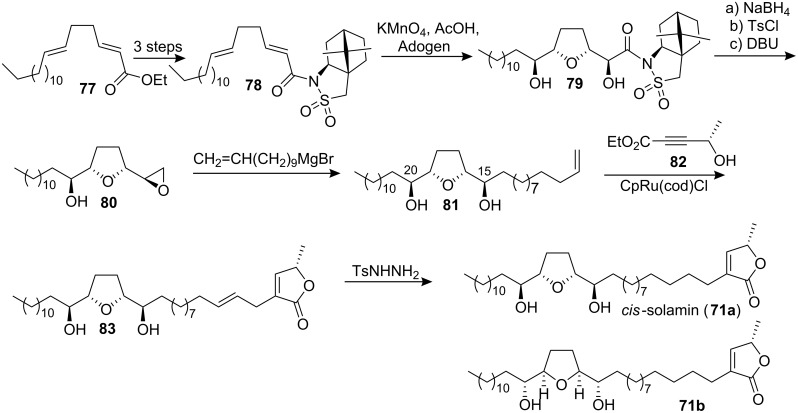
Total synthesis of *cis*-solamin by Brown’s group.

In 2002, Brown’s group [43] also reported the synthesis of *cis*-solamin (**71a**) and its diastereomer **71b** using the diastereoselective permanganate-promoted oxidative cyclization of 1,5-dienes to create the THF diol core (Scheme 11). Notably, no protecting groups were required during the stages of fragment assembly. The synthesis of precursor **78** for the oxidative cyclization reaction was completed by hydrolysis of **77** and activation of the resulting unsaturated acid as the acid chloride followed by reaction with lithiated (*2S*)-10,2-camphorsultam. The key oxidative cyclization reaction, conducted under phase-transfer conditions, introduced the C15, C16, C19, and C20 stereocenters present in *cis*-solamin in one step, then the auxiliary was best removed from **79** by reduction using NaBH_4_. The resulting diol was taken forward by conversion to the epoxide **80**. Addition of the C3-C13 fragment in a copper-catalyzed Grignard reaction afforded **81**. The butenolide ring in 4,5-dehydro-*cis*-solamin (**83**) was put in place using a ruthenium catalyzed Alder-ene reaction of **81** with **82**. Final selective reduction of the 4,5-double bond completed the synthesis of **71a**. The diastereomeric structure **71b** was also synthesized following the same route but using the (2*R*)-10,2-camphorsultam. In 2004, the full details of this total synthesis were reported [44].

**Scheme 12:**
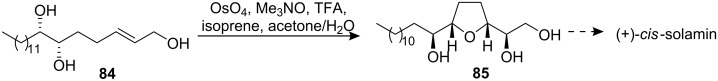
The formal synthesis of (+)-*cis*-solamin by Donohoe’s group.

In 2005, Donohoe’s group [45] reported the formal synthesis of (+)-*cis*-solamin using oxidative cyclization of diol **84** with catalytic osmium tetroxide which gave the THF product **85** in high yield (Scheme 12).

**Scheme 13:**
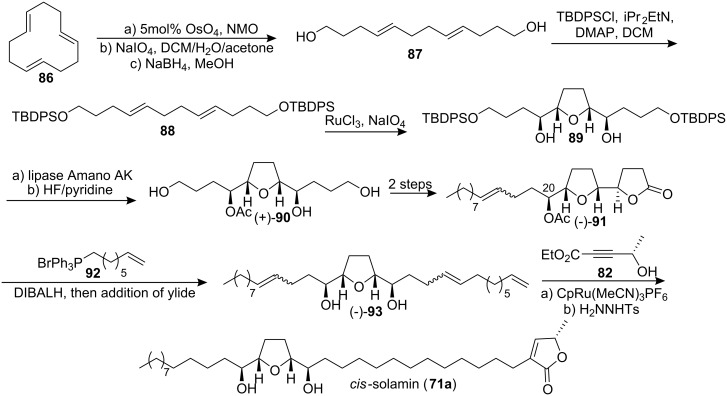
Total synthesis of *cis*-solamin by Stark’s group.

In 2006, Stark’s group [46] accomplished an enantioselective total synthesis of *cis*-solamin using a highly diastereoselective ruthenium tetroxide catalyzed oxidative cyclization as a crucial transformation (Scheme 13). In their synthetic route, commercially available (*E*,*E*,*E*)-1,5,9-cyclododecatriene **86** was readily converted into diene **87**. Standard silyl protection of diol **87** afforded the cyclization precursor **88**. Treatment of diene **88** under the ruthenium tetroxide catalyzed oxidative conditions resulted in a smooth conversion of the starting material into THF **89**. Triol (+)-**90** was obtained with lipase Amano AK desymmetrization. For the appropriate side chain attachment, the termini were differentiated to give lactone (−)-**91**. Reduction of (−)-**91** followed by a Wittig reaction yielded fully deprotected product (−)-**93**. Finally, the introduction of the butenolide segment using a ruthenium(II)-catalyzed Alder-ene reaction followed by selectively reduction furnished *cis*-solamin (**71a**). Spectroscopic data for this compound were identical to those reported for *cis*-solamin isolated from natural sources [41].

#### Total synthesis of mosin B

**Scheme 14:**
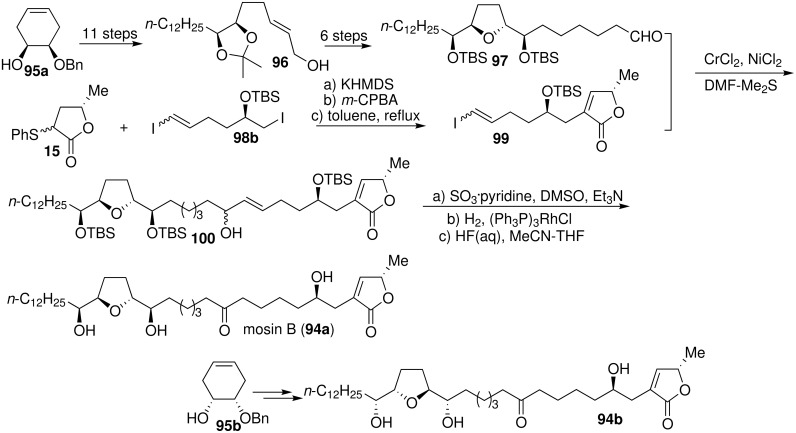
Total synthesis of mosin B by Tanaka’s group.

Mosin B (**94**) is a mono-THF acetogenin isolated by McLaughlin’s group [47] from the bark of *Annona squamosa* and shows selective cytotoxic activity against the human pancreatic tumor cell line, pancreatic cancer cells (PACA-2) (ED_50_ = 2.5 × 10^−4^ µg/mL), with a potency 100 times that of adriamycin (ED_50_ = 1.8 × 10^−2^ µg/mL) [47]. In 2001, a total synthesis of the *threo*/*trans*/*erythro*-type acetogenin mosin B (**94a**) and one of its diastereomers **94b** had been achieved by Tanaka’s group (Scheme 14) [48]. The THF core segment **97** was stereoselectively constructed by iodoetherification of *E*-allylic alcohol **96**, which was prepared from chiral alcohol **95a**. The γ-lactone segment **99** was synthesized by *α*-alkylation of *α*-sulfenyl γ-lactone **15** with **98b**. The carbon skeleton **100** was assembled in a convergent fashion from **97** and **99** through a Nozaki-Hiyama-Kishi reaction. Oxidation of **100** followed by deprotection afforded the candidate **94a**. The other candidate **94b** was synthesized from **95b** using the same procedure. Comparison of the specific rotation of synthetic **94a** and **94b** with the naturally occurring mosin B suggested that the absolute configuration of natural mosin B was **94a**.

#### Total synthesis of longicin

**Scheme 15:**
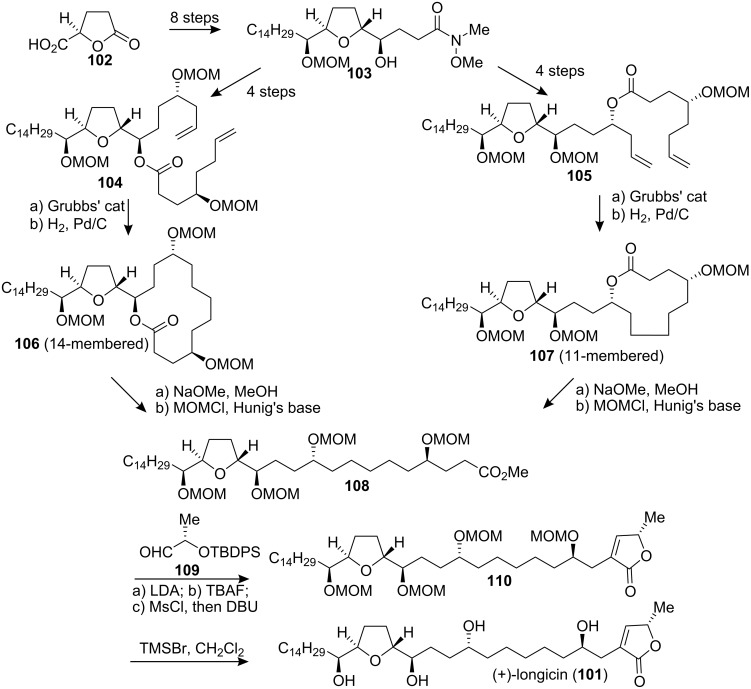
Total synthesis of longicin by Hanessian’s group.

In 1995, McLaughlin’group reported the isolation of longicin (**101**) from *Asimina longifolia* (Annonaceae). Longicin was reported to exhibit over 1 million-fold selective antitumor activity against PACA-2 (IC_50_ = 1.25 × 10^−9^ µg/mL) compared to adriamycin (IC_50_ = 1.95 × 10^−3^ µg/mL)[49]. In 2005, Hanessian’s group [50] reported the first total synthesis, stereochemical assignment, and structural confirmation of longicin (**101**) (Scheme 15). The strategy involved the use of Grubbs’ RCM reaction as a “chain elongation” strategy for the synthesis of acetogenin-type structures and a new protocol for butenolide incorporation. Prepared from D-glutamic acid, lactone **102** was converted to the desired *threo*-*trans*-*erythro* THF isomer **103**, which could be converted to the different two diolefins **104** and **105**. The 14- (**106**) and 11-membered (**107**) ring lactones were obtained by an ester-tethered RCM macrocyclization. Hydrogenation of the olefins followed by saponification of macrolactones **106** and **107** with NaOMe and subsequent MOM protection gave the common intermediate **108** with identical physical data independent of the route used. The lithium enolate formed from **108** with LDA was treated with **109** and the resulting aldol product was desilylated and in situ lactonization gave **110**. Removal of the MOM groups afforded longicin (**101**), which was identical to the natural product on the basis of the reported physical constants [49].

#### Total synthesis of murisolin

**Scheme 16:**
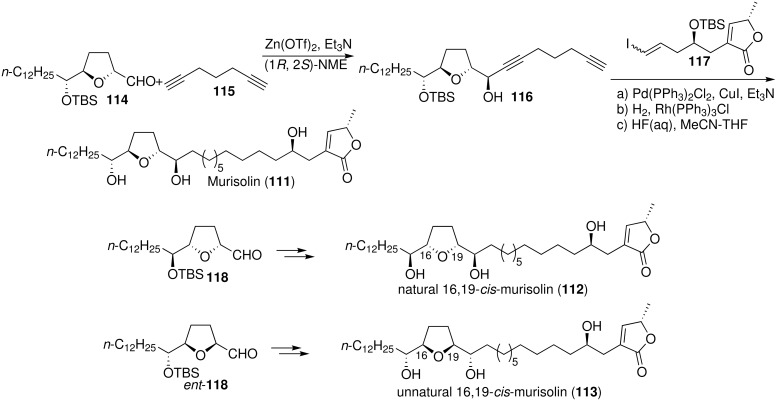
Total synthesis of murisolin and 16,19-*cis*-murisolin by Tanaka’s group.

Murisolin (**111**) is a mono-THF acetogenin, isolated from the seed of *Annona muricata* by Cortes’s group [51], which shows selective cytotoxic activity against human lung carcinoma (A-549) (ED_50_ = 5.90 × 10^−8^ µg/mL), human colon adenocarcinoma (HT-29) (ED_50_ = 6.58 × 10^−8^ µg/mL), and human kidney carcinoma (A-498) (ED_50_ = 1.09 × 10^−9^ µg/mL) with potency from 10^5^ to 10^6^ times that of adriamycin (ED_50_ = 3.99 × 10^−3^ µg/mL for A-549, ED_50_ = 2.43 × 10^−2^ µg/mL for HT-29 and ED_50_ = 2.26 × 10^−3^ µg/mL for A-498) [52]. In 2004, Tanaka’s group [53] reported the first total synthesis of murisolin (**111**) (Scheme 16). The *threo*/*trans*/*threo*-type THF ring moiety **116** was constructed with excellent stereoselectivity by asymmetric alkynylation of α-tetrahydrofuranic aldehyde **114** with 1,6-heptadiyne (**115**). Then Sonogashira coupling of **116** and the iodide **117** followed by hydrogenation and deprotection provided murisolin (**111**). The spectroscopic data of synthetic **111** (^1^H NMR, ^13^C NMR, IR, MS, mp) were in good agreement with those reported. In 2005, the full details of this total synthesis [54] were reported, along with the total synthesis of natural 16,19-*cis*-murisolin **112** and unnatural 16,19-*cis*-murisolin **113** from **118** and *ent*-**118** respectively using a similar procedure.

**Scheme 17:**
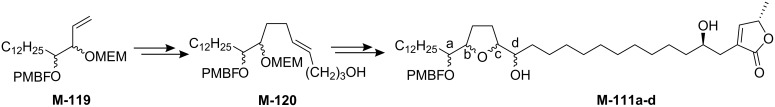
Synthesis of a stereoisomer library of (+)-murisolin by Curran’s group.

In 2004, Curran’s group [55] reported a 4-mix/4-split strategy for the synthesis of a stereoisomer library of (+)-murisolin and 15 of its isomers, which relied on solution phase technique of fluorous mixture synthesis (Scheme 17). In their synthetic route, a single mixture of M-**119**, which was tagged with different fluorous PMB tags, was transformed into alkene M-**120** and was then followed by two splits. First, each of the two mixtures was subjected to a Shi epoxidation with enantiomeric ketone catalysts. Later, these two mixtures were split again, half being subjected to a Mitsunobu reaction and the other half not. Ultimately, they obtained four mixtures M-**111a**–**d**, each containing four isomers, which were demixed and detagged to provide all 16 target isomers. In 2006, the full details of this work were described [56].

**Scheme 18:**
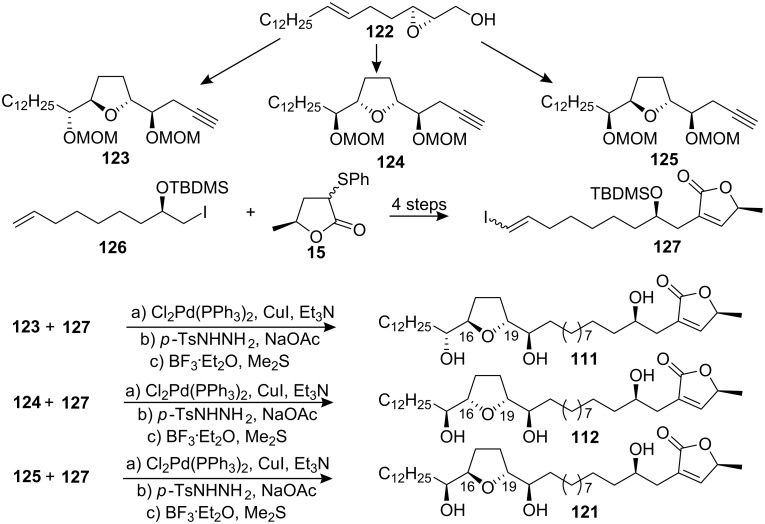
Total synthesis of murisolin by Makabe’s group.

In 2006, Makabe’s group [57] reported the total synthesis of murisolin (**111**), (15*R*,16*R*,19*S*,20*S*)-*cis*-murisolin (**112**), and (15*R*,16*R*,19*R*,20*S*)-murisolin A (**121**) (Scheme 18). The mono-THF moieties were synthesized from epoxy alcohol **122** by using Sharpless AD-mix-β for the *threo*-*trans*-*threo* THF moiety **123**, a AD-mix-β followed by the Mitsunobu reaction for the *erythro-cis*-*threo* THF moiety **125**, and AD-mix-α for *threo*-*trans*-*threo* THF moiety **124**. The α,β-unsaturated γ-lactone segment **127** was synthesized through alkylation of lactone **15** and iodide **126**. The THF moiety **123** and γ-lactone **127** were coupled by a Sonogashira cross-coupling reaction to afford **111**. The syntheses of **112** and **121** were carried out as described for **111**.

#### Total synthesis of reticulatain-1

**Scheme 19:**
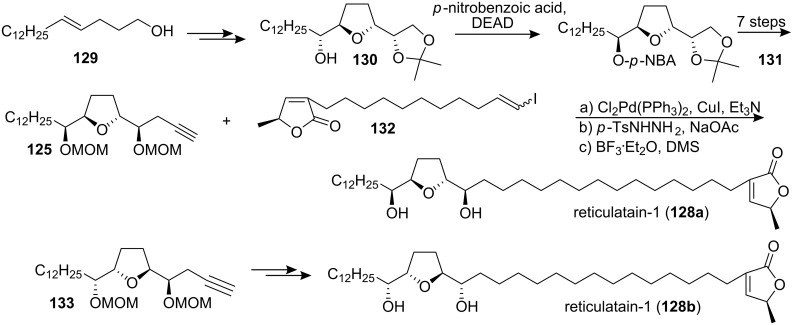
Total synthesis of reticulatain-1 by Makabe’s group.

Reticulatain-1 (**128**) is a mono-THF acetogenin, isolated from *Annona reticulata* in 1995 [58]. To determine the absolute configuration of natural **128**, Makabe’s group [59] reported the total synthesis of **128a** and **128b** in 2004 (Scheme 19). **130** was obtained by using the Sharpless epoxidation and dihydroxylation of **129**. Compound **130** was then subjected to the Mitsunobu inversion to afford **131**, which was transformed into **125**. Then the THF moiety **125** and γ-lactone moiety **132** were coupled by a Sonogashira cross-coupling, and diimide reduction followed by deprotection allowed completion of the total synthesis of candidate **128a**. The other candidate **128b** was synthesized from **133** using the same procedure as that employed for **128a**. However, both of the specific rotation of synthetic **128a** ([α]_D_^30^ +9.68, *c* 1.00, CHCl_3_) and **128b** ([α]_D_^27^ +2.34, *c* 1.00, CHCl_3_) are lower than the reported value of natural occurring reticulatain-1 ([α]_D_ +22, *c* 1, CHCl_3_) [58], so comparison of the specific optical rotations of **128a** and **128b** did not allow for the strict determination of the absolute configuration. So the structure of natural reticulatain-1 has not been confirmed.

#### Total synthesis of muricatetrocin

**Scheme 20:**
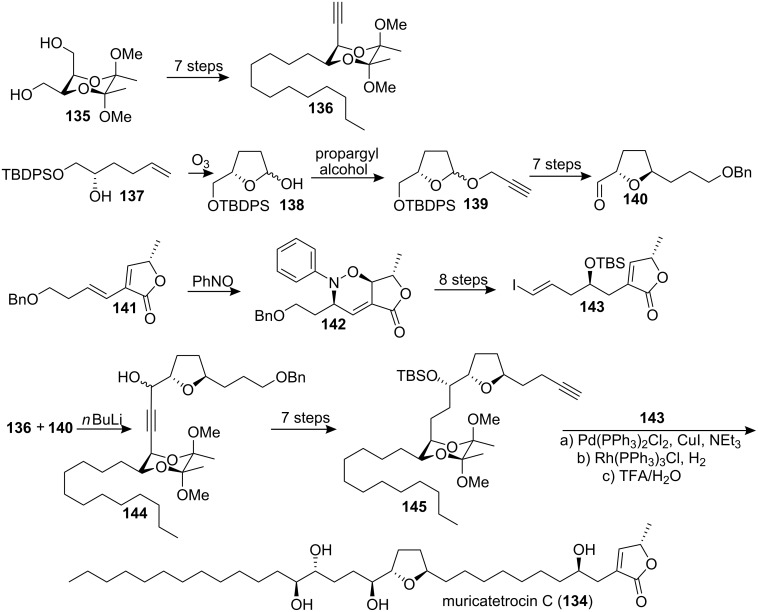
Total synthesis of muricatetrocin C by Ley’s group.

In 1996 McLaughlin’s group reported the isolation of muricatetrocin C (**134**) from the leaves of *Rollinia mucosa* [60]. The molecule exhibited potent inhibitory action against prostatic adenocarcinoma (PC-3) (ED_50_ = 1.35 × 10^−7^ µg/mL), PACA-2 (ED_50_ = 5.69 × 10^−7^ µg/mL), and A-549 (ED_50_ = 5.55 × 10^−6^ µg/mL) [60]. In 2000, Ley’s group [61] reported the first total synthesis of muricatetrocin C (**134**) (Scheme 20). The *anti*-1,2-diol component **136** was obtained through selective chemical differentiation of the hydroxyl termini in diol **135**. The 2,5-*trans*-disubstituted THF unit **140** was then constructed by ozonolysis of the alkenol **137** to give the lactol **138**. Treatment of **138** with an excess of propargyl alcohol afforded **139**, which was followed by anomeric O-C rearrangement to give **140**. The hetero-Diels-Alder (HDA) reaction between diene **141** and nitrosobenzene followed by N-O bond cleavage and elimination of the aryl amine to reintroduce the butenolide unsaturation afforded **143**. Then coupling reaction between **136** and **140** provided **144**, which was readily transformed into **145**. Sonogashira cross-coupling of **145** with the vinyl iodide **143** followed by selective hydrogenation, desilylation and removal of the butane diacetal group finished the total synthesis of **134**. The spectroscopic data for synthetic **134** (^1^H NMR, ^13^C NMR, IR, MS, mp and specific rotation) were in excellent agreement with those reported for naturally occurring muricatetrocin C (**134**). In 2002, the full details of this work were described [62].

**Scheme 21:**
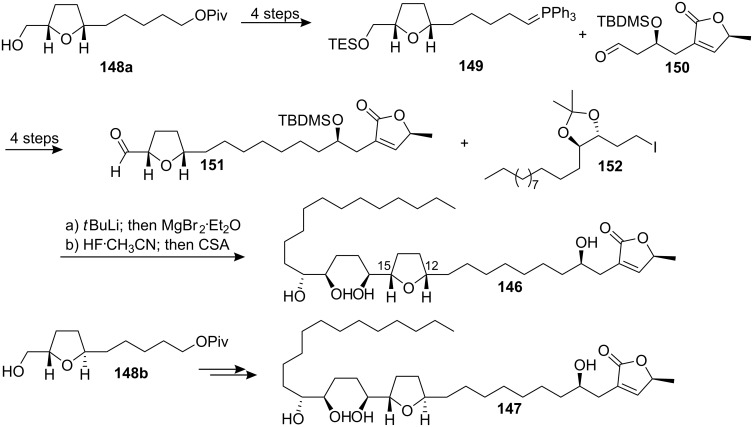
Total synthesis of (4*R*,12*S*,15*S*,16*S*,19*R*,20*R*,34*S*)-muricatetrocin (**146**) and (4*R*,12*R*,15*S*,16*S*,19*R*,20*R*,34*S*)-muricatetrocin (**147**) by Koert’s group.

In 1993 McLaughlin’s group reported the isolation of two new mono-THF acetogenins from *Annona muricata* [33]. They were named muricatetrocin A and B. In 1994 Yang’s group published analytical data for howiicin E isolated from *Goniothalamus howii*, which indicated a constitutional identity and a stereochemical match for muricatetrocin A and howiicin E [63]. In 2000, Koert’s group [64] reported the total synthesis of (4*R*,12*S*,15*S*,16*S*,19*R*,20*R*,34*S*)-muricatetrocin (**146**) and (4*R*,12*R*,15*S*,16*S*,19*R*,20*R*,34*S*)-muricatetrocin (**147**) based on a modular synthetic strategy which had been used in the total synthesis of mucocin (Scheme 21) [65]. The ylide **149**, which was synthesized from the *cis*-THF alcohol **148a**, was coupled with the butenolide aldehyde **150** *via* a Wittig reaction to afford the THF aldehyde **151** after further 3 steps. Then addition of the magnesium derivative of iodide **152** to aldehyde **151** followed by global deprotection provided compound **146**. The compound **147** was synthesized from the *trans*-THF alcohol **148b** following a similar procedure. Compound **146** showed analytical data in agreement with howiicin E and a fit with the data for muricatetrocin A if one reassigns the reported ^13^C signals for C(13) and C(14). Compound **147** matched muricatetrocin B in respect to all NMR data. However, a lower optical rotation was found for **147** ([α]_D_^28^ = +6.7) than was reported for the natural product ([α]_D_^25^ = +15.0).

### 2 Adjacent bis-THF ACGs

The core unit of the adjacent bis-THF acetogenins contains six oxygenated stereocenters, and much of the synthetic work on the family has been focused in that direction. The first successful approach was recorded in 1991 by Hoye’s group who employed a two-directional inside-out epoxide cascade sequence to prepare a core enantiomer of uvaricin [66]. This synthesis was important in establishing the absolute stereostructure of the natural product. Subsequently, numerous synthetic approaches to related core THF arrays have been reported.

#### Total synthesis of parviflorin

**Scheme 22:**
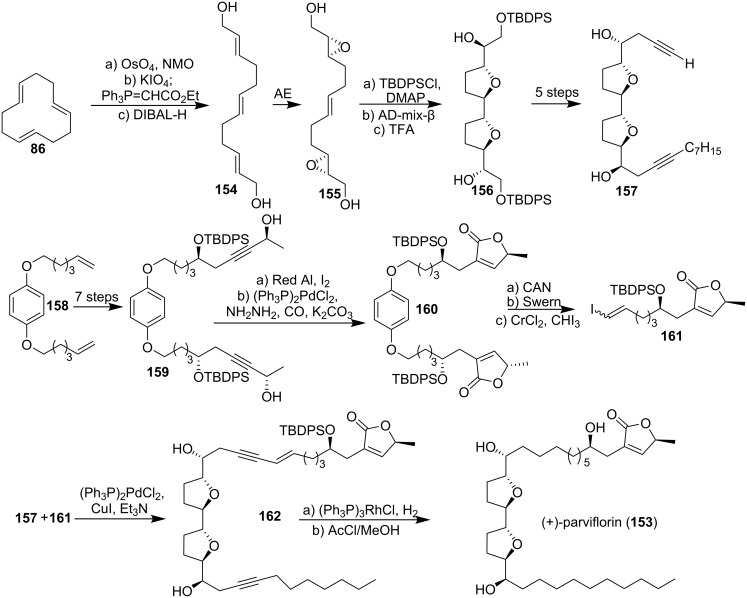
Total synthesis of parviflorin by Hoye’s group.

Parviflorin (**153**), a relatively rare C_35_ adjacent bis-THF acetogenin, was isolated by McLaughlin’s group both from *Asimina parviflora* [67] and from *Annona bullata* [68]. Parviflorin showed remarkable selectivity in its cytotoxicity against certain human solid tumor cell lines [67–68]. In 1996, Hoye’s group [69] achieved the first synthesis of parviflorin (**153**) by using a highly efficient construction of the adjacent bis-THF backbone (Scheme 22). 1,5,9-Cyclododecatriene (**86**) was converted to the bis-allylic alcohol **154** through selective oxidation and Wittig extension followed by DIBAL-H reduction. The stereogenic centres in the bis-THF backbone **156** were then installed by sequential double Sharpless AE/Sharpless AD. The building block **157** was then constructed through bidirectional chain synthesis strategy. The propargylic alcohol **159**, obtained from 1,4-bis(alkenyloxy)benzene **158**, was converted into butenolide **160** through Red-Al reduction, iodine treatment, and carbonylation. Oxidative release of **160** followed by Swern oxidation and Takai reaction provided the terminal vinyl iodide **161**. Final Pd^0^-catalyzed coupling of alkyne **157** with vinyl iodide **161** gave the enediyne **162**, which underwent selective hydrogenation and desilylation to give (+)-parviflorin (**153**).

**Scheme 23:**
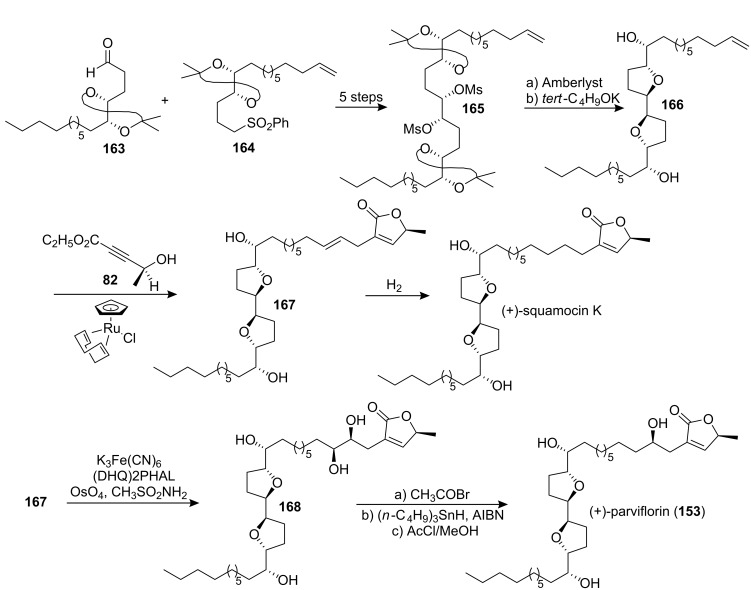
Total synthesis of parviflorin by Trost’s group.

In 1997, Trost’s group [70] reported the total synthesis of (+)-parviflorin (**153**) through a flexible approach (Scheme 23). The bis-acetonide **165**, which was constructed from the aldehyde **163** and **164**, was hydrolyzed and then treated with base to give the bis-THF **166**. Then Ru-catalyzed Alder–ene type reaction of **166** with **82** yielded the butenolide **167**. Hydrogenation of the double bond afforded (+)-squamocin K, while diastereoselective dihydroxylation of the same double bond yielded (5*S*)-hydroxyparviflorin **168**. Chemoselective deoxygenation of the C-5 hydroxyl group afforded parviflorin, spectroscopically identical to the natural product.

#### Total synthesis of trilobacin

**Scheme 24:**
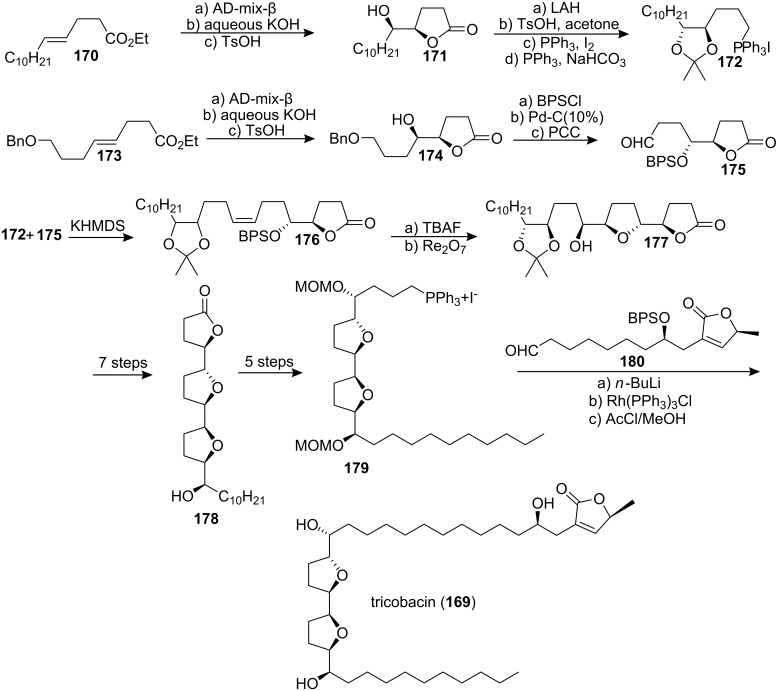
Total synthesis of trilobacin by Sinha’s group.

Trilobacin (**169**), the first known member of the adjacent bis-THF ACGs with a *threo*, *trans*, *erythro*, *cis*, *threo* backbone, was isolated from the the bark of *Asimina triloba* by McLaughlin’s group. Studies with human solid-tumor cell lines showed that trilobacin (**169**) (ED_50_ <10^−15^ µg/mL) was over 1 billion times more potent against HT-29 than adriamycin (ED_50_ = 6.69 × 10^−3^ µg/mL) [71–72]. In 1996, Sinha’s group [73] reported the first total synthesis of **169** (Scheme 24). The phosphonium salt **172** and aldehyde **175** were prepared using the AD reaction from alkenes **170** and **173**, respectively. Coupling of the Wittig reagent **172** with the aldehyde **175** produced the alkene **176**. Oxidative cyclization with Re_2_O_7_/lutidine afforded the corresponding *trans*-substituted tetrahydrofuran **177**. Alternatively, Mitsunobu inversion of the free alcohol within **177**, prior to its activation and ring-closure, gave lactone **178**, which was converted to the primary Wittig salt **179**. Treatment of **179** with BuLi and then with aldehyde **180** followed by hydrogenation and deprotection afforded **169**, which was found to be identical (^1^H NMR, [α]_D_, IR, MS) to the naturally occurring trilobacin.

#### Total synthesis of annonin I and asiminacin

**Scheme 25:**
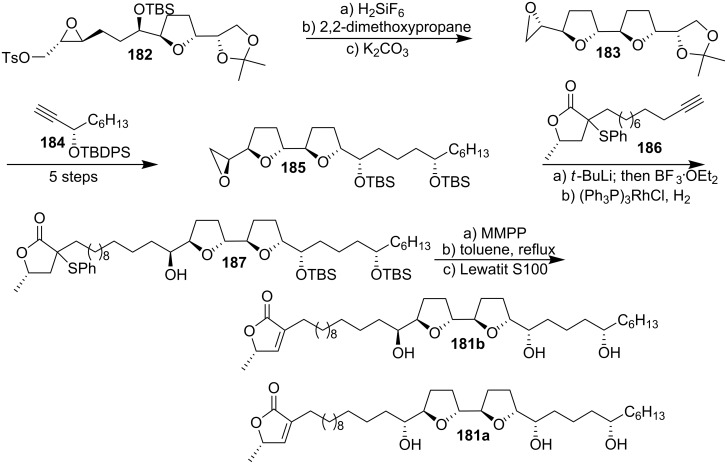
Total synthesis of 15-*epi*-annonin I **181b** by Scharf’s group.

Among the 19 ACGs that were isolated from *Annona squamosa* Born’s group found annonin I (**181a**) [74] to be the most potent compound concerning cytotoxic and insecticidal activity. With the aim to prepare interesting substances for biological assays, Scharf’s group reported the first total synthesis of 15-*epi*-annonin I (**181b**) in 1996 (Scheme 25) [75]. Ring opening – ring closing epoxide cascade [66] was performed on **182** in the presence of hexafluorosilicic acid. This produced the bis-THF moiety **183**. Opening of epoxide **183** with alkyne **184** followed by epoxide formation at the other end of the molecule afforded **185**, which was attached to lactone **186** by another epoxide opening, which produced the main structure **187**. Finally, formation of the butenolide followed by removal of the silyl protecting groups finished the total synthesis of 15-*epi*-annonin I (**181b**).

**Scheme 26:**
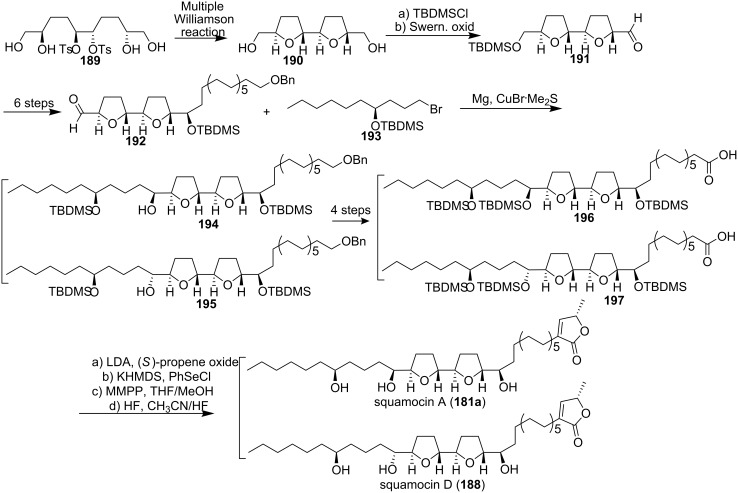
Total synthesis of squamocin A and squamocin D by Scharf’s group.

Squamocin A (**181a**) [76] (also called annonin I [74]) and squamocin D (**188**) [77] (also called asiminacin [78]) belong to a subclass of ACGs with an adjacent bis-THF subunit and an extra hydroxy group in the left side chain (C-28). Both natural products show remarkable cytotoxic activity and are interesting antitumor candidates [76–77]. In 1999, Koert’s group reported the total synthesis of these two natural products (Scheme 26) [79]. The bis-THF core of **190** with the relative *threo*-*trans*-*threo* configuration was constructed by an established multiple Williamson reaction on **189**. Monoprotection of **190** followed by oxidation gave the aldehyde **191**, which could readily be converted into the aldehyde **192**. The bromide **193** was transformed into the corresponding Grignard reagent, which was allowed to react with the aldehyde **192** to afford the two epimers **194** and **195**. The dianion of **196** was allowed to react with (*S*)-propene oxide, and subsequent deprotection of the three silyl ethers gave the target compound squamocin A (**181a**). Along the same route squamocin D (**188**) was obtained from **197**. The spectrocopic data for squamocin A and squamocin D matched the literature data. In 2000, the full details of this synthetic work were reported [80].

#### Total synthesis of asiminocin

**Scheme 27:**
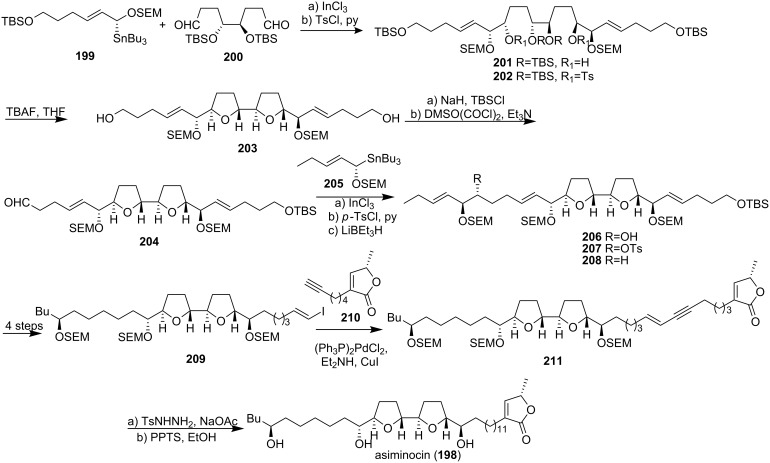
Total synthesis of asiminocin by Marshall’s group.

McLaughlin’s group published a report on the isolation and structure elucidation of asiminocin (**198**), a C_37_ ACG with nearly one billion times the cytotoxic potency of a standard reference, adriamycin, as measured against breast carcinoma (MCF-7) (ED_50_ <10^−12^ µg/mL compared to adriamycin, ED_50_ = 1.76 × 10^−2^ µg/mL) [78,81]. In 1997, Marshall’s group reported the total synthesis of asiminocin (**198**) through a bidirectional approach starting from the (*S*,*S*)-tartrate derived dialdehyde **200** and the (*R*)-α-OSEM stannane **199** (Scheme 27) [82]. Addition of **199** to **200** in the presence of InCl_3_ afforded the bis-adduct, *anti*-diol **201**. The derived tosylate **202** was converted to the bis-THF core unit **203** upon treatment with TBAF. Oxidation to aldehyde **204** followed by InCl_3_-promoted addition of the (*S*)-allylic stannane **205** gave the *anti* adduct **206**. Removal of the OH group by reduction of the tosylate **207** with LiBEt_3_H yielded the SEM ether **208**. Conversion to the vinyl iodide **209** followed by Pd^0^-catalyzed coupling with the (*S*)-alkynyl butenolide **210** gave the asiminocin precursor **211**. Selective hydrogenation of the enyne moiety with diimide and cleavage of the SEM protecting groups completed the synthesis of triol **198**, which exhibited ^1^H and ^13^C NMR spectra indentical to those of asiminocin [81].

#### Total synthesis of asiminecin

**Scheme 28:**
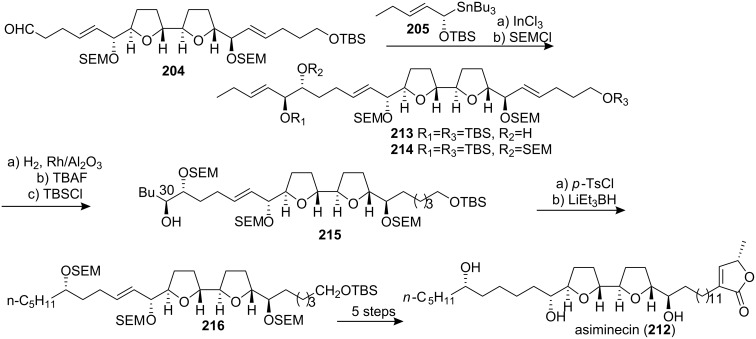
Total synthesis of asiminecin by Marshall’s group.

In 1997, Marshall’s group reported the total synthesis of asiminecin (**212**) starting from aldehyde **204** and the OTBS allylic stannane **205** (Scheme 28) [82]. Addition of the latter to the former in the presence of InCl_3_ afforded the *anti* adduct **213** which was protected as the SEM ether **214**. Hydrogenation followed by OTBS cleavage with TBAF and selective silylation of the primary alcohol with TBSCl and Et_3_N-DMAP led to the secondary alcohol **215**. Tosylation and hydrogenolysis with LiEt_3_BH removed the C-30 OTs group affording the SEM ether **216**. The remaining steps for the completion of total synthesis of asiminecin were carried out along the lines described for asiminocin.

#### Total synthesis of (+)-(30*S*)-bullanin

**Scheme 29:**
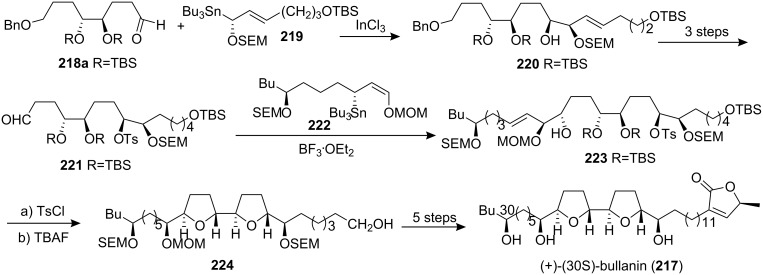
Total synthesis of (+)-(30*S*)-bullanin by Marshall’s group.

(+)-Bullanin was isolated from the stem bark of *Asimina triloba* as an inseparable mixture of 30*S* and 30*R* diastereomers [83]. In 1998, Marshall’s group reported the total synthesis of (+)-(30*S*)-bullanin (**217**) through S_E_2′ additions of oxygenated non-racemic allyl stannane (Scheme 29) [84]. Transmetallation of stannane **219** with InCl_3_ in the presence of aldehyde **218a** afforded the expected *anti*-adduct **220**. Addition of stannane **222** to aldehyde **221** in the presence of BF_3_·OEt_2_ afforded the *syn*-adduct **223** as the only detectable stereoisomer. Tosylation of the alcohol followed by exposure to TBAF promoted *bis*-THF cyclization. Introduction of the butenolide moiety finished the total synthesis of (+)-(30*S*)-bullanin (**217**). The identity of this material with that of the 30*S* natural isomer was established through ^1^H NMR comparison of the tri-(*S*)-MTPA (Mosher) ester with that of the (*S*)-Mosher ester of the mixture derived from natural sources. The optical rotation of their synthetic material, [α]_D_ +24, was in close agreement with the reported value for the mixture, [α]_D_ +28.

#### Total synthesis of uvaricin

**Scheme 30:**
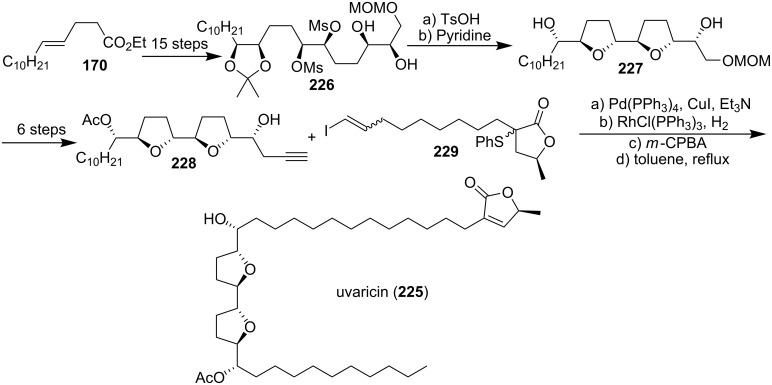
Total synthesis of uvaricin by the group of Sinha and Keinan.

Uvaricin (**225**), an adjacent bis-THF acetogenin which was isolated in 1982 from *Uvaria acuminata*, was of special historical value because it was the first ACG discovered [6]. In 1998, the group of Sinha and Keinan reported the first total synthesis of the naturally occurring isomer **225** using three consecutive Sharpless AD reactions to place the necessary oxygen functions on a “naked” carbon skeleton **226** in a regio- and enantioselectively controlled manner (Scheme 30) [85]. The appropriate bis-THF ring system **227** was constructed using a Williamson type etherification reaction on a functionalized bis-mesylate intermediate. A Sonogashira cross-coupling reaction of the terminal acetylene **228** with vinyl iodide **229** followed by hydrogenation and thermal elimination finished the total synthesis of **225**. ^1^H NMR and ^13^C NMR data were found to be identical to the reported spectral data.

**Scheme 31:**
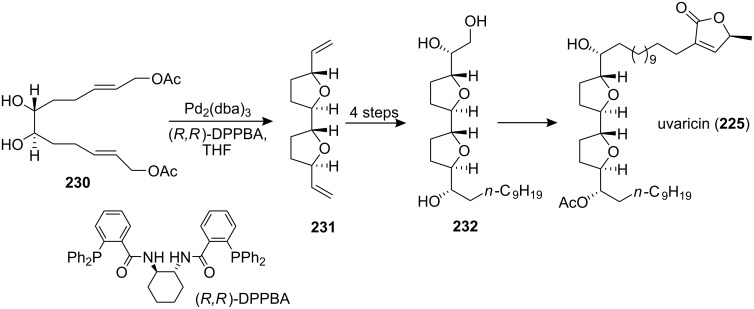
Formal synthesis of uvaricin by Burke’s group.

In 2001, Burke’s group reported the synthesis of a known intermediate **232** in the synthesis of uvaricin (**225**) (Scheme 31) [86]. A chiral DPPBA ligand controlled double cyclization of **230** allowed the selective formation of a single diastereomer **231** in one step, thus providing general access to annonaceous acetogenins containing *trans*/*threo*/*trans* or *cis*/*threo*/*cis* bis-THF core structures. Desymmetrization of diene **231** with AD-mix-β provided the known triol **232**, which has served as an intermediate in a total synthesis of uvaricin (**225**).

#### Total synthesis of trilobin

**Scheme 32:**
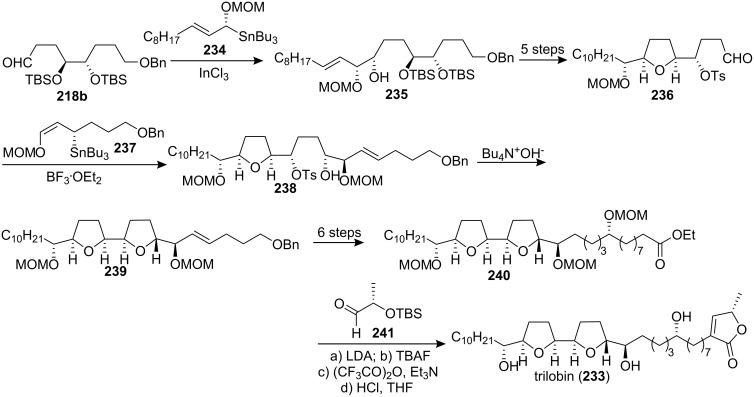
Total synthesis of trilobin by Marshall’s group.

Trilobin (**233**), which was isolated from the the bark of *Asimina triloba* by McLaughlin’s group [71] in 1992, has high potency against human lung cancer, breast cancer, and colon cancer cell lines (10^6^ to nearly 10^10^ times the cytotoxic potency of the reference compound, adriamycin) [71]. In 1999, Marshall’s group reported the total synthesis of trilobin (Scheme 32) [87]. Addition of the γ-alkoxy allylic indium reagent derived from the (*R*)-α-OMOM allylic stannane **234** and InCl_3_ to aldehyde **218b** afforded the *anti*-adduct **235** as a single diastereomer, which could be cyclized to the *cis*-*threo*-THF **236**. Completion of the bis-THF core was effected by addition of the (*S*)-γ-OMOM allylic stannane **237** to aldehyde **236**, affording the *syn* adduct **238**. Treatment of this adduct with Bu_4_NOH led to the bis-THF **239**. Introduction of the butenolide moiety was achieved through condensation of ester **240** with a protected lactic aldehyde, which afforded product **233**, identified as (+)-trilobin through comparison of the ^1^H and ^13^C NMR spectra with those of the natural product.

**Scheme 33:**
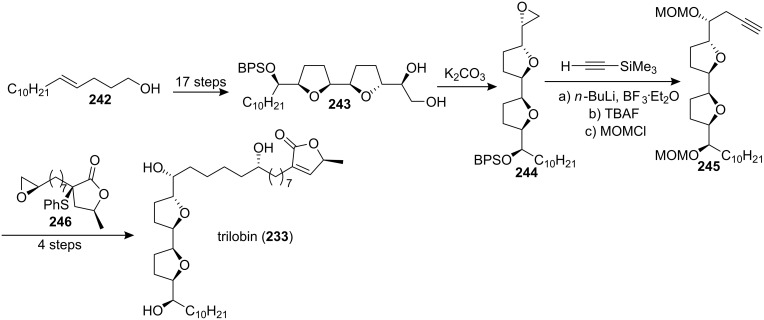
Total synthesis of trilobin by the group of Sinha and Keinan.

At the same time, the group of Sinha and Keinan also reported the first total synthesis of **233** using the different “naked” carbon skeleton strategy [88], with all of the asymmetric centers in the bis-THF fragment **243** being produced by the Sharpless AD and AE reactions, starting with alcohol **242** (Scheme 33). Then the reaction of epoxide **244** with trimethylsilylethynyllithium and subsequently with the butenolide precursor **246** finished the total synthesis of trilobin. Synthetic trilobin (**233**) and its *R*- and *S*-Mosher’s esters showed ^1^H NMR data identical to those of the naturally occurring trilobin and its *R*- and *S*-Mosher ester derivatives.

#### Total synthesis of asimilobin

**Scheme 34:**
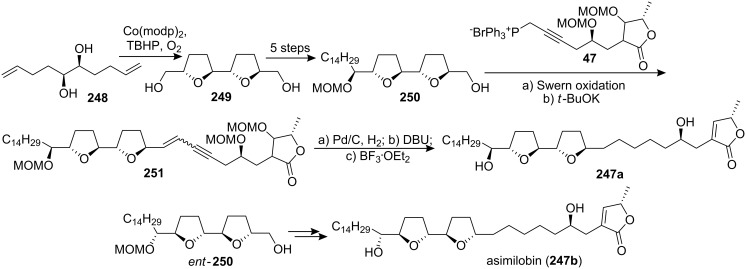
Total synthesis of asimilobin by the group of Wang and Shi.

Asimilobin (**247**) was isolated by McLaughlin’s group, both from the seeds of *Asimina triloba* [89] and from the bark of *Goniothalamus giganteus* (Annonaceae) [90], and showed cytotoxicity values comparable with adriamycin against six human solid-tumor cell lines [89–90]. In 1999, the group of Wang and Shi reported the first total synthesis of asimilobin (Scheme 34) [91]. Compound **248** was smoothly oxidized and cyclized to form a *C*_2_-symmetrical bis-THF compound **249** using Co(modp)_2_ as a catalyst under an oxygen atmosphere. Mono-protection of the diol **249** followed by Swern oxidation, and then reaction of the resulting aldehyde with CH_3_(CH_2_)_13_MgCl gave the bis-THF segment **250**. The coupling reaction between the aldehyde prepared from **250** and the ylide prepared from **47** gave the enyne **251**, which was hydrogenated. Global deprotection allowed completion of the synthesis of **247a**. The spectral data (^1^H and ^13^C NMR, HRMS) of the synthetic compound **247a** were consistent with those reported for the title compound in literature. However, the specific rotation was opposite to that reported. {Synthetic compound **247a**: [α]_D_^20^ −11.4 (*c* 0.70, CHCl_3_), [α]_D_^26^ −11.9 (*c* 0.43, CH_2_Cl_2_); Lit. [90] [α]_D_ +6.0 (*c* 0.05, CHCl_3_); Lit. [91] [α]_D_ +11.3 (*c* 1.00, CH_2_Cl_2_)}. In order to clarify this problem, they immediately synthesized diastereomer **247b** using the enantiomer of segment **250** made via the same procedures. They found that **247b** had the same spectral data and close specific rotation as that reported in literature. {[α]_D_^24^ +6.4 (*c* 0.36, CHCl_3_); [α]_D_^25^ +7.0 (*c* 0.10, CH_2_Cl_2_)}. Thus, this work strongly suggested that the natural product had the opposite absolute configuration on the bis-THF unit to that reported in the literature. In 2000, the full details of this total synthesis were reported [92].

#### Total synthesis of squamotacin

**Scheme 35:**
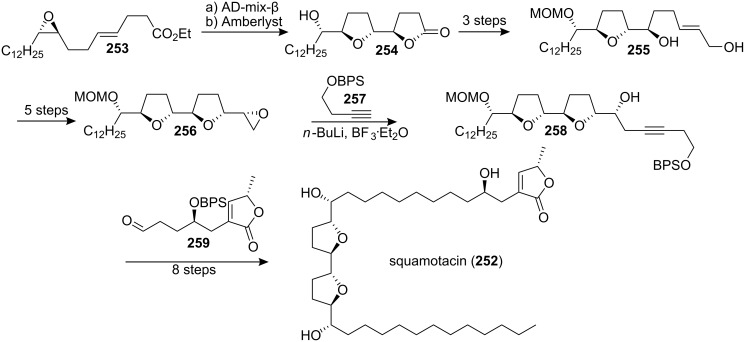
Total synthesis of squamotacin by the group of Sinha and Keinan.

Squamotacin (**252**), which was isolated from the bark of *Annona squamosa*, showed cytotoxic selectivity for PC-3 (ED_50_ = 1.72 × 10^−9^ µg/mL), with a potency of over 10^8^ times that of adriamycin (ED_50_ = 3.42 × 10^−1^ µg/mL) [93]. Its structure had been proposed on the basis of ^1^H and ^13^C NMR, MS, and IR spectral data [93]. In 1999, the group of Sinha and Keinan reported the first total synthesis of (+)-squamotacin (**252**) [94] through a “naked” carbon skeleton strategy where all asymmetric centers in the bis-THF fragment of the molecules **256** were produced by the Sharpless AD and AE reactions (Scheme 35). Elongation of the carbon skeleton of **256** was achieved by a ring-opening reaction using **257** to afford alkyne **258**. Then Wittig reaction of the corresponding Wittig reagent prepared from **258** with aldehyde **259** followed by catalytic hydrogenation and deprotection afforded **252**. The synthetic compound **252** was found to be identical, by ^1^H and ^13^C NMR, and MS, with the naturally occurring squamotacin.

#### Total synthesis of asimicin

**Scheme 36:**
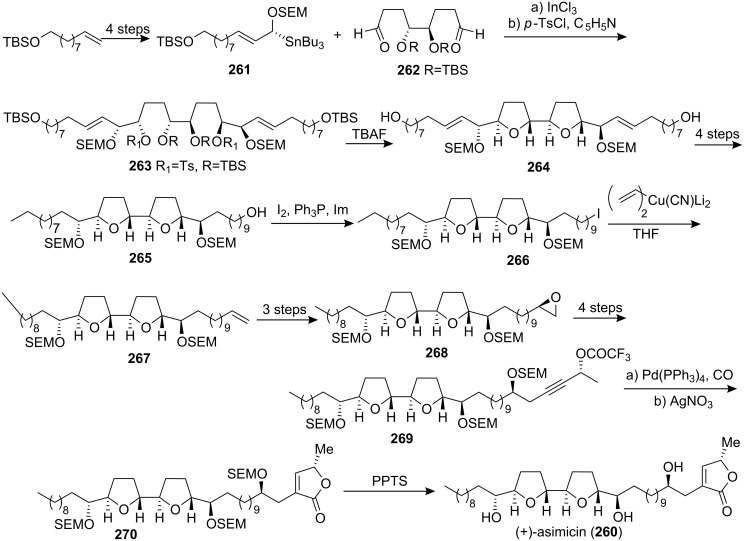
Total synthesis of asimicin by Marshall’s group.

Asimicin (**260**), which was isolated from the pawpaw tree, *Asimina triloba* [95], was synthesized by Marshall’s group in 1997 [96]. The approach employed the (*R*)-α-OSEM allylic stannane **261** reaction with the dialdehyde **262** obtained from (*S*,*S*)-diethyl tartrate to afford the bis-adduct **263** (Scheme 36). Treatment of **263** with TBAF led to the core bis-THF intermediate, diol **264**. Mono tosylation and subsequent hydrogenolysis with LiBEt_3_H gave alcohol **265**. The iodide **266** was coupled with the higher-order vinylcyanocuprate to afford olefin **267**, which could be converted to the epoxide **268**. Addition of (*R*)-lithio-2-(OTBS)-3-butyne afforded the trifluoroacetate **269**, then **269** was converted into the butenolide **270**. Cleavage of the SEM protecting group afforded (+)-asimicin (**260**). The ^1^H and ^13^C NMR spectra and optical rotation of the synthetic **260** were identical to those reported for (+)-asimicin, [α]_D_ 15.0 (*c* 0.2, CHCl_3_), reported [α]_D_ 14.7 (*c* 0.3 CHCl_3_) [95].

**Scheme 37:**
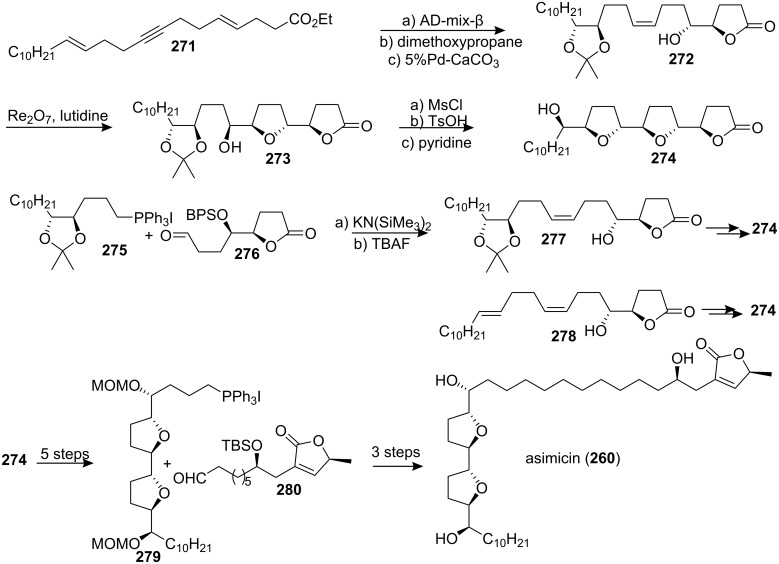
Total synthesis of asimicin by the group of Sinha and Keinan.

In 2000, the group of Sinha and Keinan reported the total synthesis of asimicin (**260**) [97] and demonstrated the advantages of three different strategies for the synthesis of the tricyclic intermediate **274** (Scheme 37), which represented the key fragment of the bis-THF ACGs. The naked carbon skeleton strategy was based on the production of all asymmetric centers by selective placement of the oxygen functions onto an unsaturated, non-functionalized carbon skeleton **271**. Diversity in this approach arose from the relative timing of highly stereoselective reactions, such as the Sharpless AD reaction and the Kennedy oxidative cyclization (OC) with rhenium(VII) oxide. The convergent strategy, which was based on the combinatorial coupling of two series of diastereomeric fragments **275** and **276**, to produce intermediate **277**, enjoyed the advantages of both efficiency and versatility. The third approach, which was based on partially functionalized intermediates, such as **278**, combined the advantages of both the linear and the convergent strategies, synthetic efficiency and diversity. The phosphonium salt **279**, which was synthesized from **274**, was reacted with aldehyde **280** in a Wittig reaction, which, after global deprotection, allowed completion of the total synthesis of asimicin (**260**). The spectral data (^1^H and ^13^C NMR) of synthetic asimicin was identical to those of naturally occurring compounds.

**Scheme 38:**
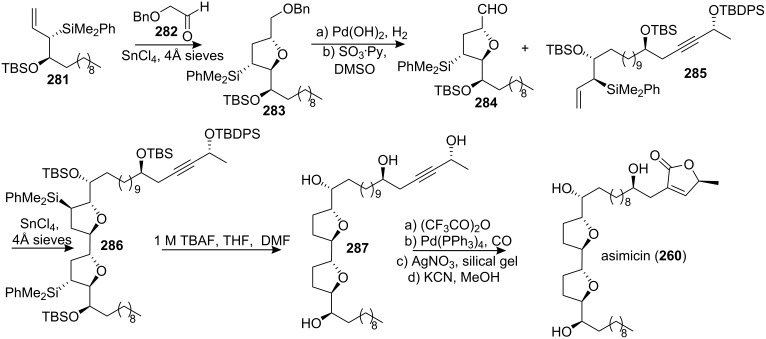
Total synthesis of asimicin by Roush’s group.

In 2005, Roush’s group synthesized the bis-THF core of asimicin [98] from two sequential chelate-controlled [3+2] annulation reactions of allylsilanes and appropriately substituted aldehydes (Scheme 38). Subjecting the protected allylsilane **281** to the [3+2] annulation reaction with *α*-benzyloxyacetaldehyde (**282**) afforded the 2,5-*trans*-THF **283**. Conversion of **283** to aldehyde **284** was achieved by reductive removal of the benzyl group and subsequent oxidation of the alcohol. Treatment of aldehyde **284** with allylsilane **285** mediated by SnCl_4_ afforded the bis-THF **286**. Finally, the butenolide ring was installed using a procedure developed by Marshall’s group [96] to provide synthetic (+)-asimicin.

**Scheme 39:**
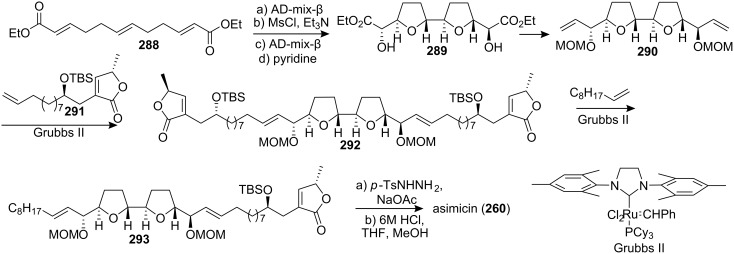
Total synthesis of asimicin by Marshall’s group.

In 2006, Marshall’s group reported the total synthesis of asimicin by a highly convergent route in which Grubbs cross-metathesis played a key role (Scheme 39) [99]. The bis-THF core unit **289** was constructed through a bidirectional outside-in hydroxy mesylate cascade cyclization route from **288**. The bisbutenolide analogue **292** was prepared from diene **290** and the butenolide segment **291** through Grubbs cross-metathesis. Reaction of the bisbutenolide **292** with 1-decene catalyzed by Grubbs II catalyst led to the asimicin precursor **293**, which was selectively hydrogenated, and subsequent global deprotection afforded asimicin (**260**). Analogues that differed in the length of the alkyl chain were also obtained in this way.

#### Total synthesis of 10-hydroxyasimicin

**Scheme 40:**
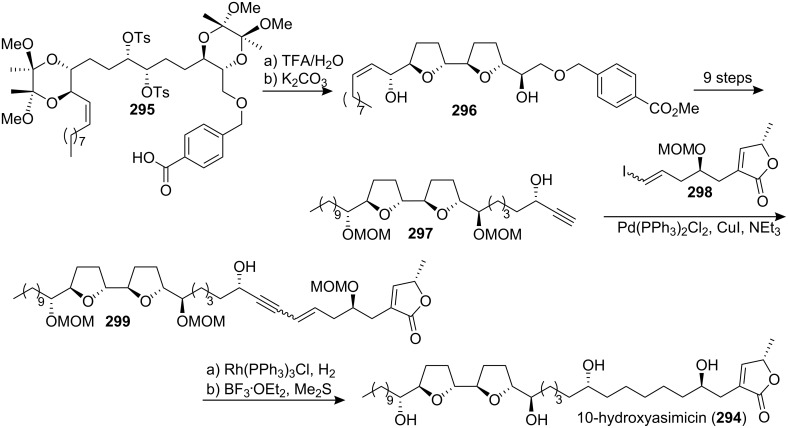
Total synthesis of 10-hydroxyasimicin by Ley’s group.

In 2005, Ley’s group reported the total synthesis of 10-hydroxyasimicin (**294**) (Scheme 40) [100]. Williamson cyclization of **295** led to the formation of the bis-THF core **296**, which could be transformed into the fragment **297** in 9 steps. Sonogashira cross-coupling of vinyl iodide **298** with the propargylic alcohol **297** proceeded smoothly to produce the skeleton **299**. The enyne functional group was reduced selectively and final global deprotection with BF_3_·Et_2_O in dimethyl sulfide afforded **294** as a colorless wax. The spectroscopic data for synthetic **294** (^1^H NMR, ^13^C NMR, IR, MS, and specific rotation) were in excellent agreement with those reported for naturally occurring 10-hydroxyasimicin (**294**) [101].

#### Total synthesis of asimin

**Scheme 41:**
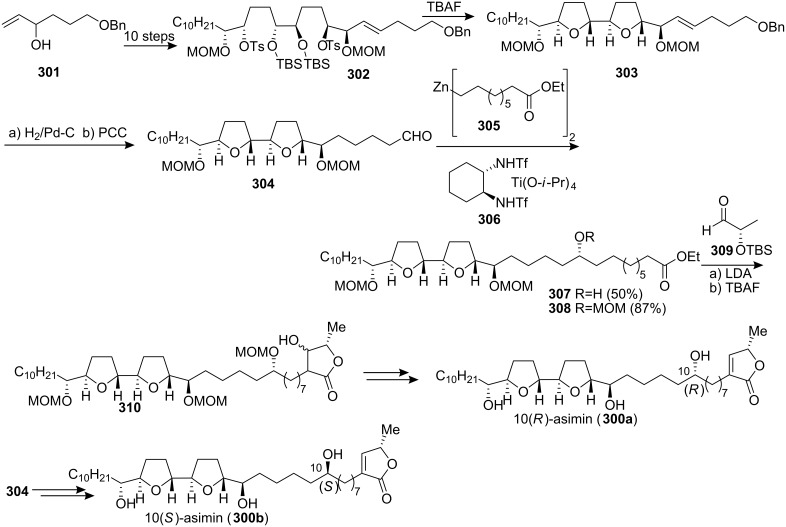
Total synthesis of asimin by Marshall’s group.

In 1994, McLaughlin’s group reported the isolation of asimin (**300**) from the stem bark of the North American paw-paw tree, *Asimina triloba* [78], depicted asimin as the C-10(*S*) isomer. However, in a subsequent paper the stereochemistry at C-10 was shown as *R* based upon chemical shift differences [81,102]. In view of the rather subtle basis for this assignment, Marshall’s group undertook a total synthesis of both C-10 epimers of asimin, reported in 1999 (Scheme 41) [103]. Their synthesis started with alcohol **301**, which was converted into bistosylate **302** in 10 stpes, then the *threo*, *trans*, *threo*, *trans*, *threo*-bis-THF core unit **303** could be obtained from **302** upon stirring with TBAF in THF. The side chain of asimin in **307** was introduced through stereoselective addition of the organozinc reagent **305** to aldehyde **304**. The ester **308** was condensed with the TBS ether of (*S*)-lactic aldehyde **309** to afford the γ-lactone adduct **310**. Exposure of the alcohol **310** to trifluoroacetic anhydride and triethylamine led to the triol **300a**. 10(*S*)-Asimin (**300b**) was prepared from aldehyde **304** by an identical sequence, using the enantiomer of **306** in the addition of the organozinc reagent. By comparing the MTPA ester of diastereomeric alcohols **300a** and **300b** with the authentic MTPA ester, the stereochemistry at C-10 of asimin (**300**) assigned as *R*.

#### Total synthesis of bullatacin

**Scheme 42:**
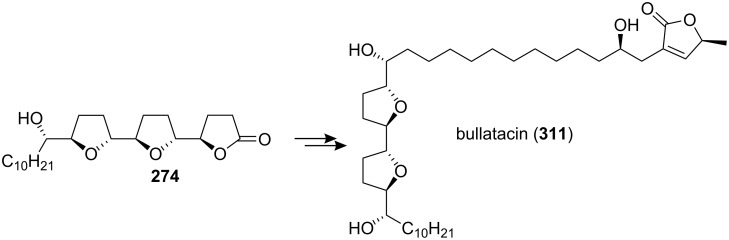
Total synthesis of bullatacin by the group of Sinha and Keinan.

In 2000, the group of Sinha and Keinan reported the total synthesis of bullatacin (**311**) [97] and demonstrated the advantages of three different strategies for the synthesis of the tricyclic intermediates **274** using the same procedure as in the total synthesis of asimicin (Scheme 42).

**Scheme 43:**
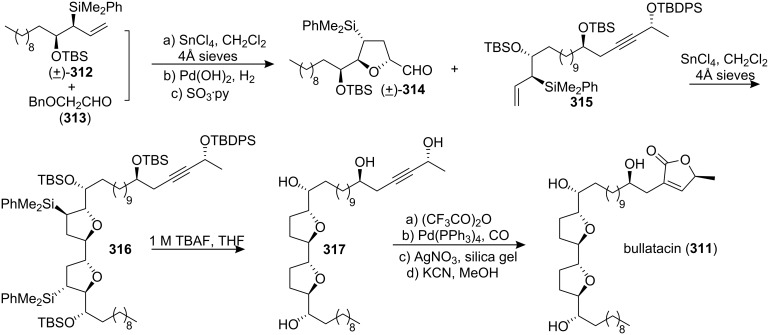
Total synthesis of bullatacin by Roush’s group.

In 2005, Roush’s group reported the total synthesis of (+)-bullatacin (**311**) *via* a diastereoselective [3+2] annulation reaction (Scheme 43) [104]. Racemic aldehyde **314**, which was prepared from allylsilane (±)-**312** and *α*-benzyloxy acetaldehyde (**313**), was treated with the highly enantiomerically enriched allylsilane **315** in the kinetic resolution manifold, providing the key bis-THF fragment **316** as a single diastereomer. Protodesilylation of the bis-THF **316** was accomplished by treatment with TBAF to give tetraol **317**. The butenolide ring was then installed completing the total synthesis of (+)-bullatacin (**311**).

**Scheme 44:**
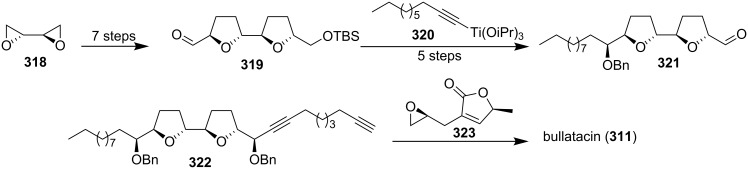
Total synthesis of bullatacin by Pagenkopf’s group.

In 2006, Pagenkopf’s group reported the total synthesis of bullatacin (**311**) in an efficient route from commercial starting materials (Scheme 44) [105]. The bis(THF) core **319** was constructed from bis-epoxide **318** through double allylation and oxidative cyclization. Then titanium acetylide **320** was reacted with bis(THF) **319** to afford **321**. Introduction of the unprotected butenolide (as **323**) by epoxide opening with lithiated **322** followed by selective reduction and deprotection afforded bullatacin.

#### Total synthesis of rollidecins C and D

**Scheme 45:**
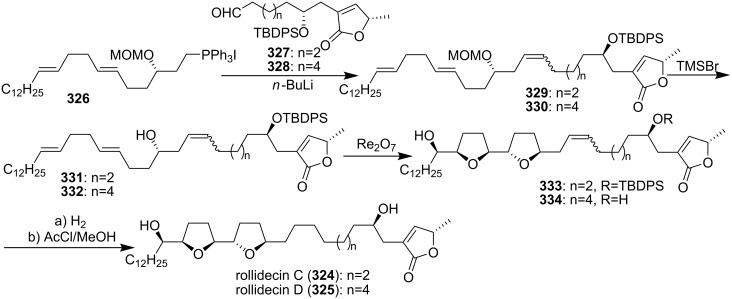
Total synthesis of rollidecins C and D by the group of Sinha and Keinan.

Rollidecin C (**324**) and rollidecin D (**325**) were discovered in the bioactive leaf extracts of *Rollinia mucosa* [106]. Both compounds **324** and **325** have exhibited cytotoxicity against six human tumor cell lines. Compound **324** was found to be uniformly more potent than **325** and showed selectivity toward HT-29 (ED_50_ = 6.26 × 10^−2^ µg/mL), exhibiting potency that approaches that of adriamycin (ED_50_ = 2.81 × 10^−2^ µg/mL for HT-29) [106]. In 2001, the group of Sinha and Keinan reported the total synthesis of rollidecins C and D (Scheme 45) [107]. Wittig reactions between the ylide derived from **326** and either of the two homologous butenolide aldehydes, **327** and **328**, produced respectively **329** and **330**, both in the form of a mixture of *E* and *Z* isomers. The MOM ether in **329** and **330** was selectively cleaved using TMSBr at low temperature, affording **331** and **332**, respectively. The oxidative biscyclization reaction was carried out with either **331** or **332** using CF_3_CO_2_ReO_3_ with trifluoroacetic anhydride (TFAA), affording the desired bis-THF products **333** or **334** respectively. Then **334** was converted to **325** by hydrogenation, and hydrogenation of compound **333** followed by desilylation afforded **324**. Both synthetic compounds **324** and **325** were found to be identical by ^1^H and ^13^C NMR with the naturally occurring rollidecins C and D, respectively.

#### Total synthesis of 30(*S*)-hydroxybullatacin

**Scheme 46:**
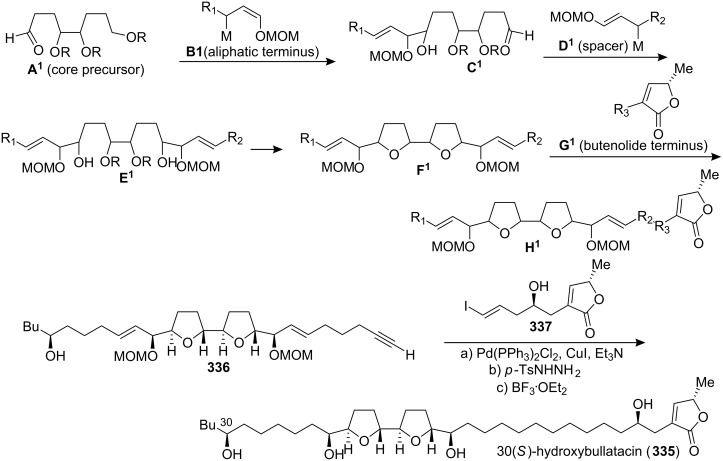
Total synthesis of 30(*S*)-hydroxybullatacin by Marshall’s group.

In 2003, Marshall’s group disclosed a modular synthetic approach to the adjacent bis-THF rings (Scheme 46) [108]. This approach featured highly selective additions of chiral *R*-oxygenated allylic stannane and indium reagents such as **B^1^** and **D^1^** (M = SnBu_3_ or InBr_2_) to an acylic core aldehyde precursor (**A^1^** then **C^1^**) followed by core ring closure (**E^1^** → **F^1^**) and ensuing Sonogashira coupling (**F^1^** + **G^1^** → **H^1^**) to append the butenolide segment. This straightforward strategy permitted the efficient assembly of the acetogenin structure from four basic subunits. By interchanging these subunits a variety of natural acetogenins and their isomers should be accessible in relatively few steps. They extended the scope of their modular four-component synthesis of annonaceous acetogenins to 30(*S*)-hydroxybullatacin (**335**). The ^1^H and ^13^C NMR spectra of the tetraol product **335** were in complete agreement with the reported spectra [109].

#### Total synthesis of uvarigrandin A

**Scheme 47:**
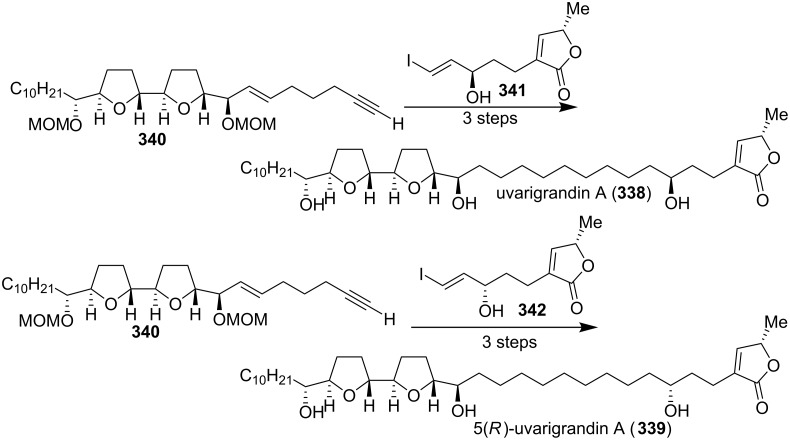
Total synthesis of uvarigrandin A and 5(*R*)-uvarigrandin A by Marshall’s group.

In 2003, Marshall’s group extended the scope of their modular four-component synthesis of ACGs to uvarigrandin A (**338**), and 5(*R*)-uvarigrandin A (**339**) (Scheme 47) [108].

#### Total synthesis of membranacin

**Scheme 48:**
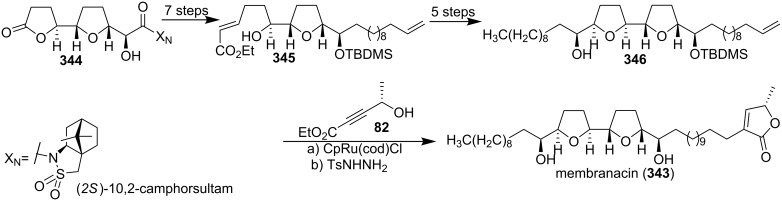
Total synthesis of membranacin by Brown’s group.

Membranacin (**343**), a cytotoxic anti-tumor acetogenin isolated from the seeds of the fruit tree *Rollinia mucosa* [110–111] was synthesized by Brown’s group in 2004 (Scheme 48) [112]. The bis-THF precursor **346** was constructed from the lactone **344** using metal-oxo and metal-peroxy-mediated oxidative cyclisations as the key steps. The butenolide portion of membranacin (**343**) was introduced using Trost’s ruthenium-catalysed Alder-ene reaction, and afforded compound **343** whose spectroscopic data were consistent with those of membranacin [110].

**Scheme 49:**
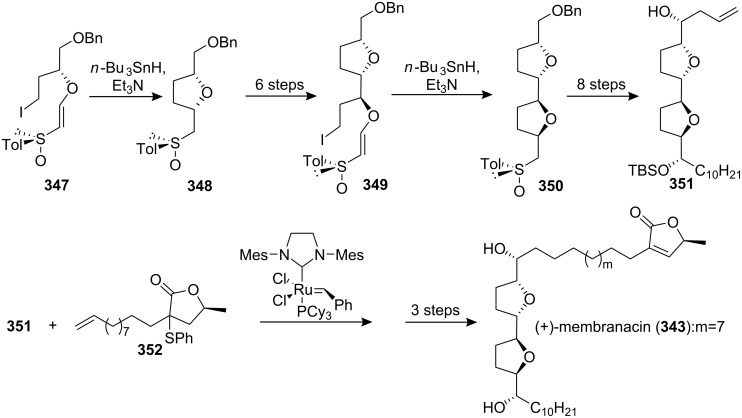
Total synthesis of membranacin by Lee’s group.

In 2005, Lee’s group also reported the total synthesis of membranacin (**343**) (Scheme 49) [113]. Radical cyclization of **347** proceeded stereoselectively to give *cis*-2,5-disubstituted oxolane product **348**, which was converted into (*E*)-β-alkoxyvinyl (*S*)-sulfoxide **349**. Radical cyclization proceeded uneventfully to yield bis-oxolane **350** in high yield. Homoallylic alcohol **351** prepared from **350** could serve as a pivotal intermediate for the natural products via cross metathesis reaction of its terminal olefin. A cross olefin metathesis reaction of **351** and **352** followed by the established three-step sequence finished the total synthesis of membranacin (**343**).

#### Total synthesis of rolliniastatin 1, rollimembrin

**Scheme 50:**
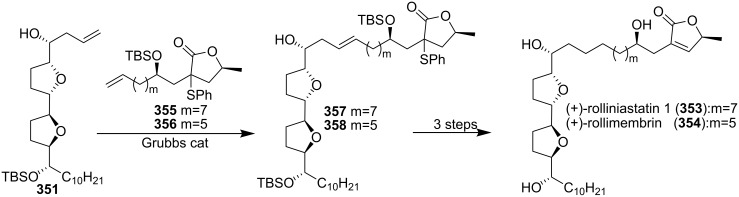
Total synthesis of rolliniastatin 1 and rollimembrin by Lee’s group.

Rolliniastatin 1 (**353**) and rollimembrin (**354**) are ACGs isolated from the seeds of *Rollinia mucosa* and *Rollinia membranacea* [9,110,114]. Lee’s group reported the first total synthesis of rollimembrin along with the total synthesis of rolliniastatin 1 in 2005 (Scheme 50) [113]. Homoallylic alcohol **351** could serve as a pivotal intermediate for the two natural products via cross metathesis reaction with their terminal olefin. A cross olefin metathesis reaction of **351** and **355** provided intermediate **357**, which was converted into rolliniastatin 1 (**353**) via the three-step sequence. Rollimembrin (**354**) was synthesized in the same manner using **356** as the metathesis partner.

#### Total synthesis of longimicin D

**Scheme 51:**
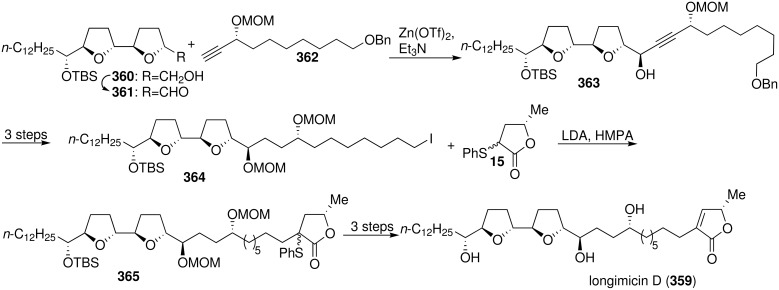
Total synthesis of longimicin D by the group of Maezaki and Tanaka.

Longimicin D (**359**), which is a structural isomer of asimicin, isolated by McLaughlin’s group from leaves and twigs of *Asimina longifolia* in 1996 [115], exhibits selective cytotoxic activities against A-549 (ED_50_ = 4.93 × 10^−4^ µg/mL), PC-3 (ED_50_ = 2.42 × 10^−4^ µg/mL), and PACA-2 (ED_50_ = 1.69 × 10^−7^ µg/mL), with potency from 10^3^ to 10^5^ times that of adriamycin [115]. In 2006, the group of Maezaki and Tanaka reported the first total synthesis of longimicin D (Scheme 51) [116]. The bis-THF alcohol **360** was oxidized to give aldehyde **361**. Introduction of the alkyne **362** into the bis-THF core **361** proceeded successfully giving the desired propargyl alcohol **363**, which was converted into iodide **364**. Alkylation of the γ-lactone **15** with the iodo-bis-THF core **364** afforded **365**. The total synthesis of longimicin D (**359**) was accomplished from **365** by subsequent reactions – (1) oxidation of the sulfide, (2) thermolytic elimination of the sulfoxide, and (3) global deprotection with acidic MeOH – to give **359** in excellent yield. The spectroscopic and physical data of synthetic **359** (^1^H NMR, ^13^C NMR, IR, MS) were in good agreement with those reported, while the specific rotation of synthetic **359** {[*α*]_D_^25^ = +23.2 (*c* 0.48 in EtOH)} was higher than that reported in the literature {[*α*]_D_ = +14 (*c* 0.1 in EtOH)}, so the structure of longimicin D needs to be further investigated.

#### Efforts toward the synthesis of mucoxin

**Scheme 52:**
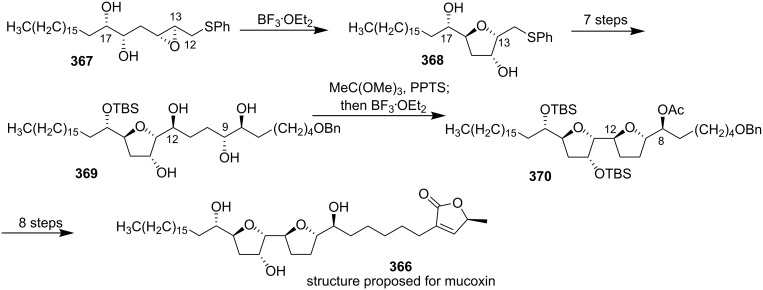
Total synthesis of the structure proposed for mucoxin by Borhan’s group.

Mucoxin (**366**), an ACG isolated from bioactive leaf extracts of *Rollinia mucosa*, was the first acetogenin containing a hydroxylated trisubstituted THF ring [117]. This natural product is a highly potent and specific antitumor agent against MCF-7 cell lines (ED_50_ = 3.7 × 10^−3^ µg/mL compared to adriamycin, ED_50_ = 1.0 × 10^−2^ µg/mL) [117]. In 2006, Borhan’s group reported the total synthesis of the proposed structure of mucoxin via regio- and stereoselective THF ring-forming strategies (Scheme 52) [118]. The 2,3,5-trisubstituted THF portion (C13-C17) **368** was accessed using a highly regioselective cyclization of epoxydiol **367**, and the 2,5-disubstituted THF ring (C8-C12) in **370** was conveniently assembled from **369** *via* a 1,2-*n*-triol cyclization strategy. The spectral data of the synthetic material did not match the reported data for the natural product. On the basis of detailed spectroscopic analysis of the synthesized molecule, they reasoned that the spectral discrepancies were due to stereochemical misassignment of the natural product. The structure of natural mucoxin need to be further revised through a different total synthesis.

#### Modular synthesis of adjacent bis-THF annonaceous acetogenins

**Scheme 53:**
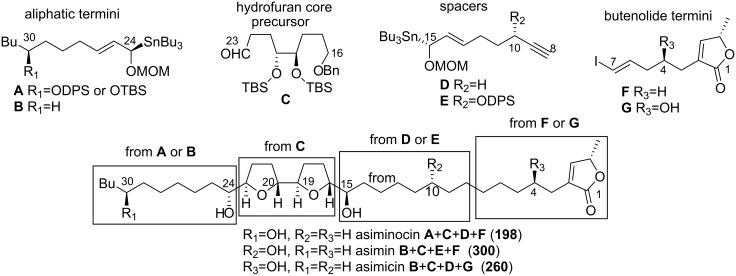
Modular synthesis of adjacent bis-THF annonaceous acetogenins by Marshall’s group.

In 2003, Marshall’s group reported a synthesis of four adjacent bis-THF ACGs, asiminocin, asimicin, asimin, and bullanin, by a modular approach from seven fundamental subunits, **A**–**G** (Scheme 53) [119]. The approach employed a central core aldehyde segment, **C**, to which were appended an aliphatic terminus, **A** or **B**, a spacer subunit, **D** or **E**, and a butenolide terminus, **F** or **G**. Coupling of the **A**, **B**, **D**, and **E** segments to the core aldehyde unit was effected by highly diastereoselective additions of enantiopure allylic indium or tin reagents. The butenolide termini were attached to the **ACD**, **BCE**, or **BCD** intermediates by means of a Sonogashira coupling. The design of the core, spacer, and termini subunits was such that any of the C30, C10, or C4 natural acetogenins or their stereoisomers could be prepared.

### 3 Nonadjacent bis-THF

#### Total synthesis of 4-deoxygigantecin

**Scheme 54:**
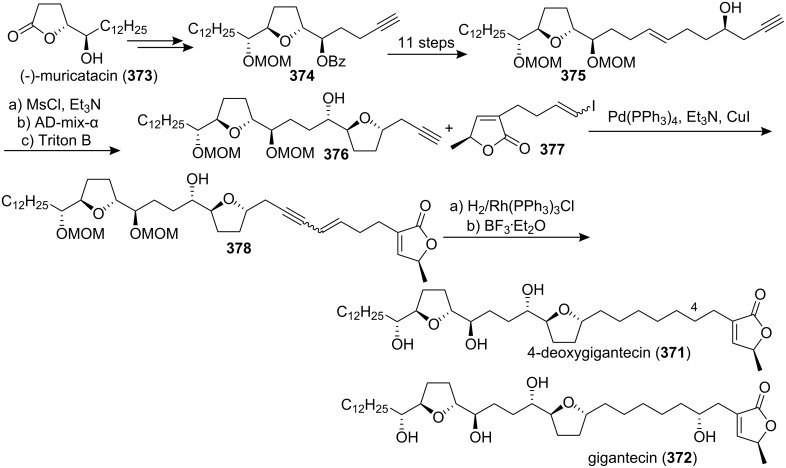
Total synthesis of 4-deoxygigantecin by Tanaka’s group.

4-Deoxygigantecin (**371**) was isolated from the bark of *Goniothalamus giganteus* by McLaughlin’s group [120]. The absolute stereochemistry of natural 4-deoxygigantecin had not yet been determined, however, it was assumed that **371** possessed, except for the C-4 carbinol center, the same absolute configuration as that of gigantecin (**372**), whose absolute stereostructure had been established by an X-ray crystallographic analysis [121]. In 1997, Tanaka’s group reported the total synthesis of natural (+)-4-deoxygigantecin (**371**) (Scheme 54) [122], which was the first example of the synthesis of a non-adjacent bis-THF type ACG. Starting with (−)-muricatacin (**373**), benzoate **374** was obtained, which was transformed into **375** in 11 steps. Mesylate formation from **375** followed by the Sharpless AD using AD-mix-α, and subsequent cyclization with Triton B furnished the key bis-THF ring-containing synthon **376**. A Pd^0^-catalyzed cross coupling reaction of compound **376** with vinyl iodide **377** gave **378**. Finally, catalytic hydrogenation of **378** and subsequent deprotection of the MOM group finished the total synthesis of (+)-4-deoxygigantecin (**371**). Its ^1^H-NMR data were in good agreement with those recorded for natural **371** and the optical rotation value {[α]_D_^23^ +16.0 (*c* 0.05, MeOH)} of the synthetic sample was also consistent with that of natural **371** {[α]_D_ +15.5 (*c* 0.2, MeOH)}. In 1998, they reported the full details of this total synthesis [123].

#### Total synthesis of squamostatin D

**Scheme 55:**
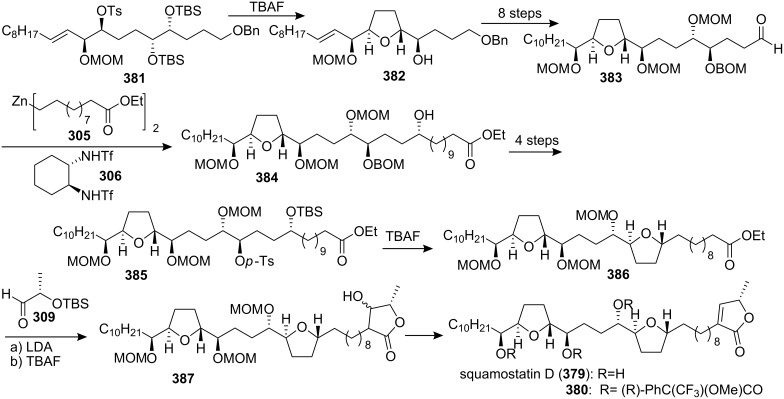
Total synthesis of squamostatins D by Marshall’s group.

In 1994, Fujimoto’s group [124] described the isolation and structure elucidation of five nonadjacent bis-THF ACGs, squamostatins A–E. In 1998, Marshall’s group reported the total synthesis of squamostatin D (**379**) (Scheme 55) [125]. The tosylate **381** was converted to the eventual *threo*,*trans*,*threo* C16-C34 segment **382** of squamostatin-D upon treatment with TBAF in THF. Introduction of the C12 stereocenter along with the C1-C11 chain **384** of squamostatin D was conveniently achieved through addition of the zinc reagent **305** to the aldehyde **383**. The derived tosylate **385** cyclized upon treatment with TBAF to afford the bis-THF ester **386**. At last, the butenolide segment of squamostatin D was introduced by a modification of the method of Wu’s group [23], affording squamostatin D (**379**), [α]_D_ +8.4 (lit. [124] +7.9), mp 112–113 °C (lit. [124] mp 112–113.5 °C), The ^1^H and ^13^C NMR spectra were identical to the spectra of the natural product as was the ^1^H spectrum of the (*R*)-Mosher ester derivative **380**.

#### Total synthesis of gigantecin

**Scheme 56:**
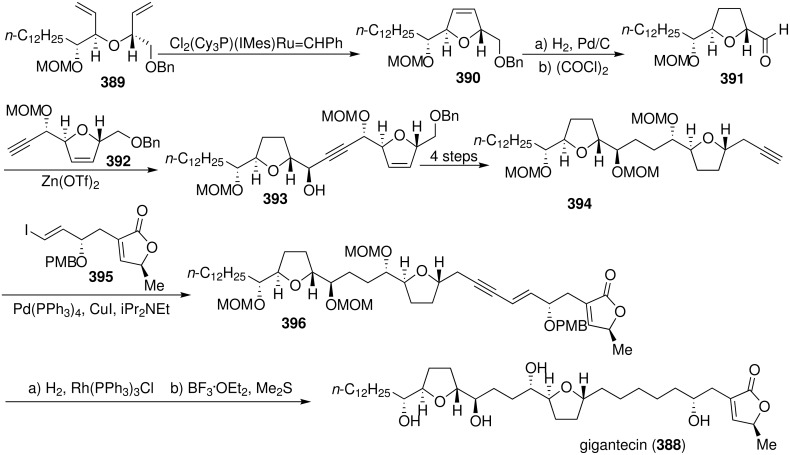
Total synthesis of gigantecin by Crimmins’s group.

Gigantecin (**388**), a representative nonadjacent bis-THF acetogenin, was isolated from the bark of *Goniothalamus giganteus* in south east Asia [126] and the seed of the Brazilian plant *Annona coriacea* [121]. The relative and absolute configurations of gigantecin were assigned after extensive spectroscopic and Mosher ester analysis, and the assignment was confirmed by single crystal X-ray analysis. Gigantecin displayed potent cytotoxicity against A-549, HT-29, MCF-7, and glioblastoma multiforme (U251MG) human tumor cell lines at ED_50_s of 0.4, 0.001, 4.3, and 0.003 µg/mL, respectively [121,126]. In 2004, Crimmins’s group reported first total synthesis of (+)-gigantecin exploiting a modified asymmetric aldol protocol using chlorotitanium enolates of oxazolidinone glycolates (Scheme 56) [127]. The diene **389** was subjected to the Grubbs catalyst resulting in formation of the dihydrofuran **390**, which was then converted to the aldehyde **391**. Addition of acetylene **392** to aldehyde **391** produced the propargylic alcohol **393**. The final C-C bond was fashioned by palladium-mediated coupling of the acetylene **394** with vinyl iodide **395** to provide enyne **396**. Selective hydrogenation followed by removal of the protecting groups led to the completion of the synthesis of (+)-gigantecin (**388**). Synthetic gigantecin was identical (^1^H, ^13^C NMR, [α]_D_^24^) to the natural material.

**Scheme 57:**
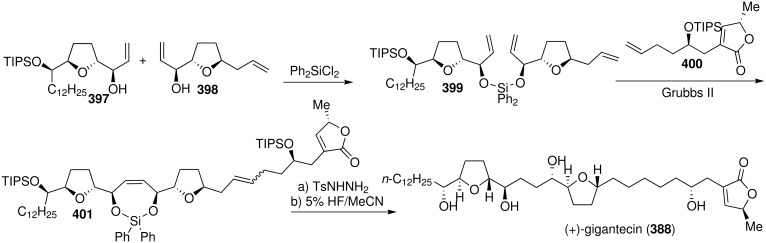
Total synthesis of gigantecin by Hoye’s group.

In 2006, Hoye’s group described an efficient, highly convergent chemical synthesis of (+)-gigantecin (**388**) utilizing a one-pot, three component olefin metathesis coupling strategy (Scheme 57) [128]. Mixed silaketal **399** was prepared by sequential loading of **397** and then **398** onto Ph_2_SiCl_2_, then triene **399** and alkene **400** were combined and exposed to Grubbs II catalyst [Ru=CHPh(Cl)_2_ (PCy_3_)(DHIMes)] to induce a ring-closing/cross-olefin methathesis sequence which afforded the major product **401**. Diimide reduction and global deprotection gave **388**.

#### Total synthesis of *cis*-sylvaticin

**Scheme 58:**
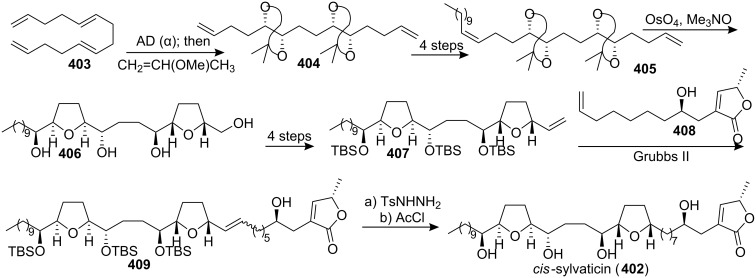
Total synthesis of *cis*-sylvaticin by Donohoe’s group.

*cis*-Sylvaticin (**402**), isolated in 1995 from the leaf extracts of *Rollinia mucosa*, was an interesting natural product with nonadjacent THF rings [129]. *cis*-Sylvaticin displays potent activity as an antitumor agent and exhibits nanomolar cytotoxicity toward human solid tumor cell lines [129]. In 2006, Donohoe’s group first reported the total synthesis of *cis*-sylvaticin (**402**) (Scheme 58) [130]. Oxidation of commercial tetradecatetraene **403** under AD conditions gave a tetraol which was immediately converted into bisacetonide **404**. The key double oxidative cyclization was then applied on bisacetonide **405** to afford the bis-THF **406**. Subsequently, the bis-THF **407** was elaborated from **406**. Then construction of **409** was accomplished by a cross metathesis reaction between **407** and **408**. The synthesis of *cis*-sylvaticin (**402**) was completed by a selective diimide reduction and acid promoted deprotection of the three OTBS groups. The synthetic material had spectroscopic data (^1^H and ^13^C NMR, [α]_D_, HRMS) identical to that reported.

### 4 Three adjacent THF rings

#### Total synthesis of goniocin

**Scheme 59:**
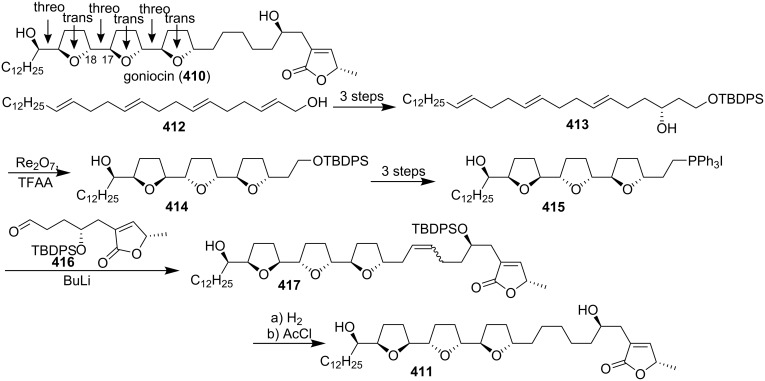
Total synthesis of 17(*S*),18(*S*)-goniocin by Sinha’s group.

Goniocin (**410**), which was isolated from *Goniothalamus giganteus* [131], possess three adjacent THF rings and, therefore, represented the first example of a new subclass of ACGs. Structure **410** was proposed for goniocin on the basis of its MS and ^1^H and ^13^C NMR data. In 1997, Sinha’s group reported that all *trans*-4,8,12-trienol substrates indeed underwent a highly stereospecific triple oxidative cyclization reaction in the presence of a rhenium(VII) reagent to produce a single stereoisomer of a tris-THF product (Scheme 59) [132]. Surprisingly, however, the product’s stereochemistry was not *trans*-*threo*-*trans*-*threo*-*trans*-*threo* as expected, but *trans*-*threo*-*cis*-*threo*-*cis*-*threo*. Consequently, they synthesized 17(*S*),18(*S*)-goniocin (**411**) rather than **410**. The key intermediate in their synthesis was the “naked” carbon skeleton **413** which was easily prepared from **412**. When trienol **413** was treated with a mixture of CF_3_CO_2_ReO_3_ and trifluoroacetic anhydride, a stereochemically pure tris-THF product **414** was obtained, which was then converted to the phosphonium salt **415**. Wittig reaction of **415** with aldehyde **416** afforded alkene **417**. Finally, hydrogenation and removal of the protecting groups afforded **411**.

**Scheme 60:**
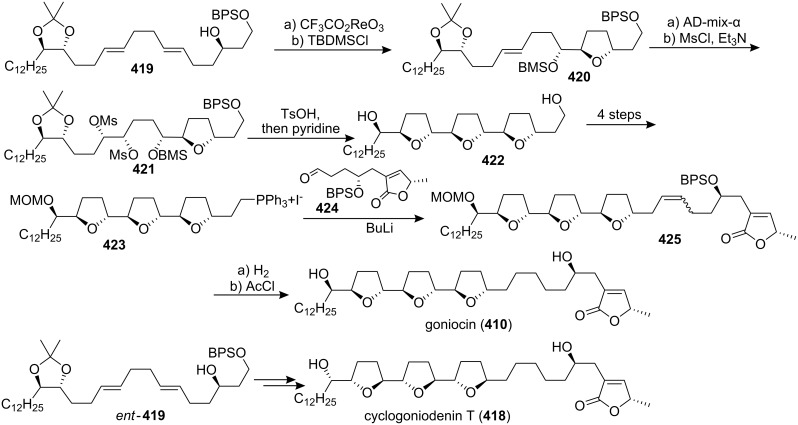
Total synthesis of goniocin and cyclogoniodenin T by the group of Sinha and Keinan.

In 1998, the group of Sinha and Keinan reported the first asymmetric total syntheses of goniocin (**410**), and cyclogoniodenin T (**418**) (Scheme 60) [133]. Oxidative cyclization of **419** with CF_3_CO_2_ReO_3_ and lutidine produced the *trans*-THF derivative **420**. Asymmetric dihydroxylation of **420** using AD-mix-α followed by double mesylation produced **421**. Acidic cleavage of the acetonide and the silyl ethers followed by heating of the resultant tetraol in pyridine produced the desired all-*trans* tris-THF diol **422**. The Wittig reagent **423** was reacted with aldehyde **424** to produce alkene **425**. Finally, catalytic hydrogenation and deprotection of both MOM and BPS groups afforded goniocin (**410**). Cyclogoniodenin T (**418**) was prepared from the *ent*-**419** using the same procedure. The absolute stereochemistry of their synthetic **410** and **418** was proved by comparison of the ^1^H NMR spectra of their (*R*) and (*S*) bis Mosher esters with the original spectra of the esters of the naturally occurring **410** and the semisynthetic **418**.

### 5 Adjacent THF-THP rings

#### Total synthesis of jimenezin

**Scheme 61:**
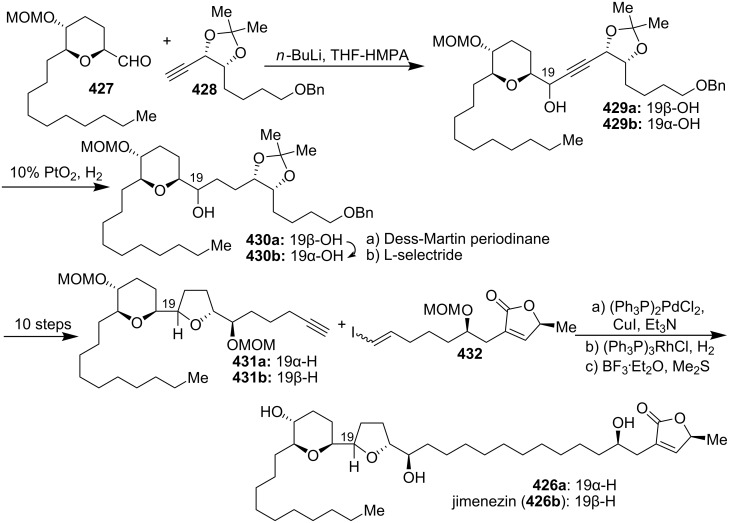
Total synthesis of jimenezin by Takahashi’s group.

In 1998, Mata’s group isolated a new ACG from the seeds of *Rollinia mucosa* (Annonaceae) and named it jimenezin (**426a**) [134]. This natural product was quite active in the BST assay (IC_50_ 5.7×10^−3^ µg/mL) [135], and exhibited potent cytotoxic activity against six human solid tumor cell lines. In 1999, Takahashi’s group reported the first total synthesis of jimenezin that dictated revision of the formula to **426b** (Scheme 61) [136]. The coupling reaction between **427** and **428** afforded a 92:8 mixture of the desired carbinol **429a** and its diastereomer **429b**. Hydrogenation of the mixture using PtO_2_ gave the desired 19β-alcohol **430a** along with its 19-epimer **430b**. Dess-Martin oxidation of the mixture (**430a** and **430b**), and subsequent reduction with L-Selectride could transform the **430a** into **430b**. The 19β-alcohol **430a** was then transformed to the central core **431a** in 10 steps. Finally, the complete carbon skeleton of **426a** was assembled by joining **431a** and **432** under Hoye’s conditions. The spectroscopic and physical properties of the synthetic material **426a** were found to differ from those of natural jimenezin, so the 19α-alcohol **430b** was transformed into the terminal acetylene derivative **431b**, which was coupled with **432** affording **426b**, whose physical and spectral data ([α]_D_^20^, ^1^H and ^13^C NMR) were identical with those of the natural jimenezin.

**Scheme 62:**
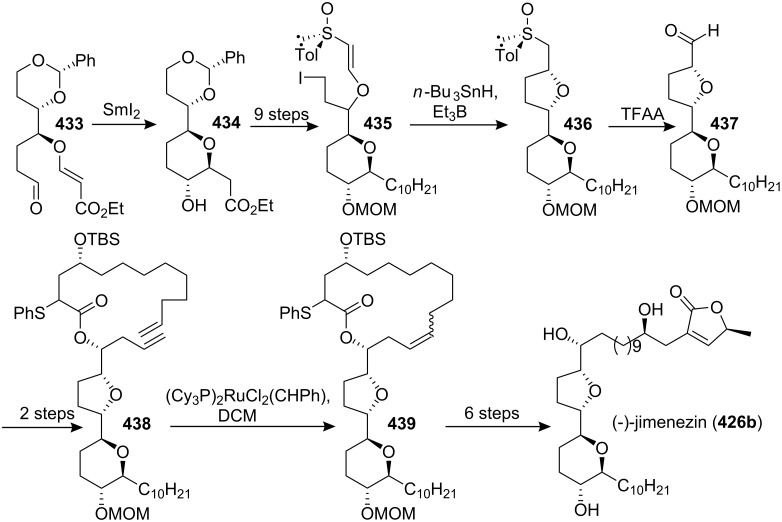
Total synthesis of jimenezin by Lee’s group.

In 2005, Lee’s group reported the total synthesis of jimenezin (**426b**) *via* radical cyclization of β-alkoxyacrylate and β-alkoxyvinyl sulfoxide intermediates and intramolecular olefin metathesis reaction (Scheme 62) [137]. Hydroxy oxane **434** was prepared from a β-alkoxyacrylate aldehyde precursor **433** *via* samarium(II) iodide-mediated cyclization. Oxolane derivative **436** was obtained *via* radical cyclization of β-alkoxyvinyl sulfoxide **435**. The homoallylic alcohol prepared from aldehyde **437** was converted into carboxylate ester **438**, which could serve as a precursor for macrolactone **439** *via* ring-closing olefin metathesis. Incorporation of an (*S*)-propylene oxide unit into **439** and further manipulations generated jimenezin (**426b**).

**Scheme 63:**
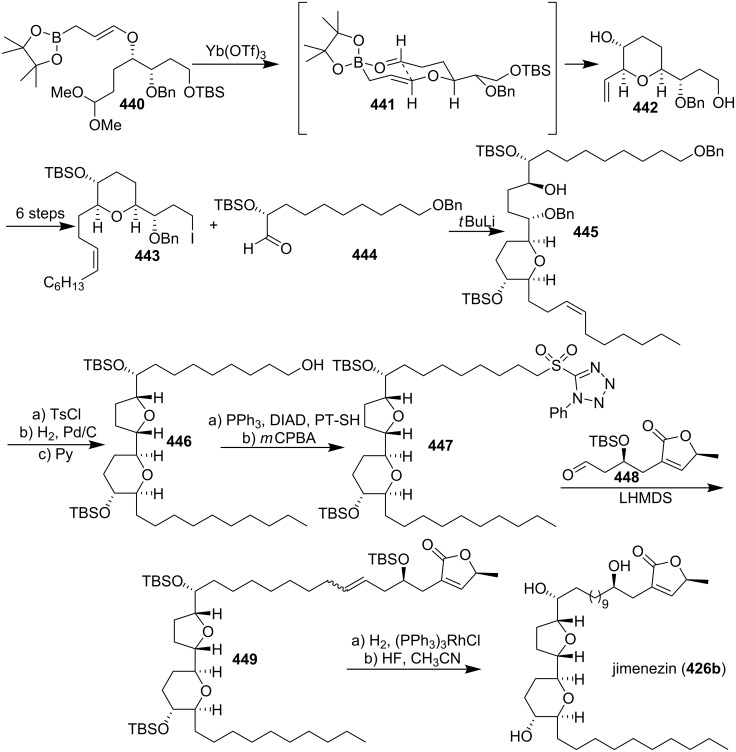
Total synthesis of jimenezin by Hoffmann’s group.

In 2006, Hoffmann’s group reported the total synthesis of jimenezin (**426b**) [138] through a highly stereoselective intramolecular allylboration to establish the tetrahydropyran ring and an intramolecular Williamson reaction to close the THF ring (Scheme 63). Treatment of the *E*-allyl boronate **440** with LiBF_4_ or Yb(OTf)_3_ in acetonitrile with 2% water led to the desired allylboration product **442**, which was transformed into compound **443**. An iodine-lithium exchange reaction of **443** gave the corresponding organolithium compound, which added to the aldehyde **444** afforded the desired stereoisomer **445**. Tosylation of the secondary hydroxy group in **445** followed by hydrogenation and refluxing in pyridine produced compound **446**. The alcohol **446** was converted into the 1-phenyl-1*H*-tetrazol-5-yl (PT) sulfone **447**. A Julia-Kocienski olefination of the sulfone **447** with the aldehyde **448** gave the alkene **449**. Chemoselective hydrogenation of the double bond in **449** followed by cleavage of all three silyl ethers provided (−)-jimenezin (**426b**), which was found to be identical to the natural product with respect to the spectroscopic data [134].

#### Total synthesis of muconin

**Scheme 64:**
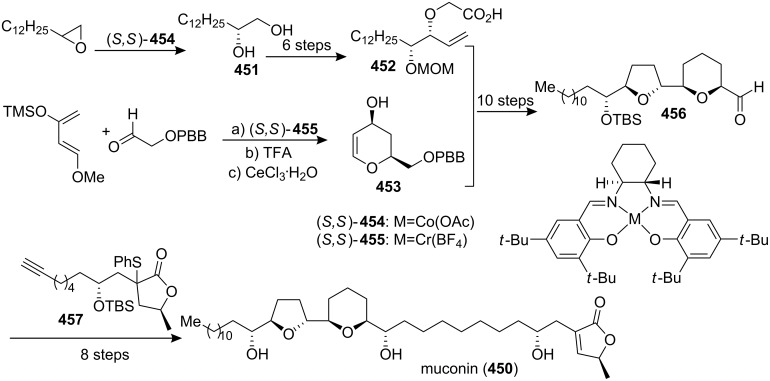
Total synthesis of muconin by Jacobsen’s group.

Muconin (**450**), which was isolated from the leaves of *Rollinia mucosa* by McLaughlin’s group in 1996 [117], has exhibited potent and selective in vitro cytotoxicity against PACA-2 (ED_50_ = 5.4 × 10^−4^ µg/mL) and MCF-7 (ED_50_ = 2.4 × 10^−4^ µg/mL) in a panel of six human solid tumor cell lines [117]. In 1998, Jacobsen’s group reported the total synthesis of muconin through a chiral building block approach (Scheme 64) [139]. The hydrolytic kinetic resolution (HKR) of (±)-tetradeceneoxide using complex (*S*,*S*)-**454** afforded (*R*)-tetradecane-1,2-diol **451**, which could be converted to acid **452**. Pyranol **453** was constructed by the hetero-Diels-Alder condensation of 1-methoxy-3-[(trimethylsilyl)oxy]-1,3-butadiene with *p*-bromobenzyloxyacetaldehyde catalyzed by (*S*,*S*)-**455**. Esterification of **453** with acid **452** followed by ring-closing metathesis and further elaboration afforded **456**. Coupling of **457** with aldehyde **456** followed by elimination and deprotection finished the total synthesis of **450** which exhibited spectral properties identical to those of the natural product.

**Scheme 65:**
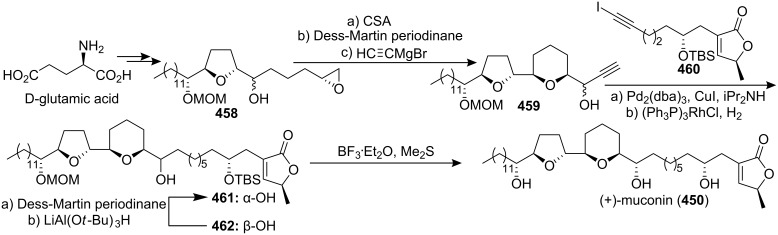
Total synthesis of (+)-muconin by Kitahara’s group.

In 1999, Kitahara’s group also reported the total synthesis of (+)-mucocin (**450**) (Scheme 65) [140]. The epoxide **458**, which was constructed from the D-glutamic acid, could be transformed into **459**. Palladium(0)-mediated coupling of the alkyne **459** with the iodoalkyne **460** followed by hydrogenation afforded **461** and **462**, and the undesired β-alcohol **462** was inverted to α-alcohol **461** by means of a Dess-Martin oxidation/LiAl(O*t*-Bu)_3_H reduction sequence. Finally, global deprotection provided (+)-muconin (**450**) with spectral properties identical to those of the natural product [117]. In 2000, they reported the full details of this total synthesis [141].

**Scheme 66:**
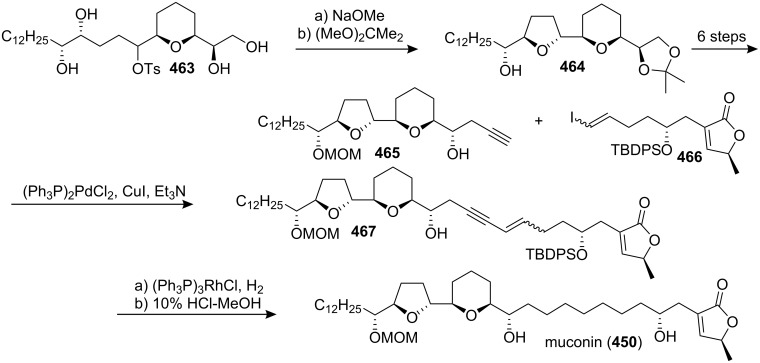
Total synthesis of muconin by Takahashi’s group.

In 2002, Takahashi’s group reported the total synthesis of muconin (**450**) through a coupling reaction of a THF–THP segment and a terminal butenolide (Scheme 66) [142]. The cyclic ether **464**, which was obtained by heating **463** with sodium methoxide in methanol, could be transformed into a terminal acetylene **465**. Then the complete carbon skeleton of **450** was assembled by joining **465** and **466** under Hoye’s conditions to give enyne **467**, which underwent regioselective reduction and deprotection to give muconin (**450**). The spectroscopic and physical properties of **450** were identical those of natural **450** [117].

**Scheme 67:**
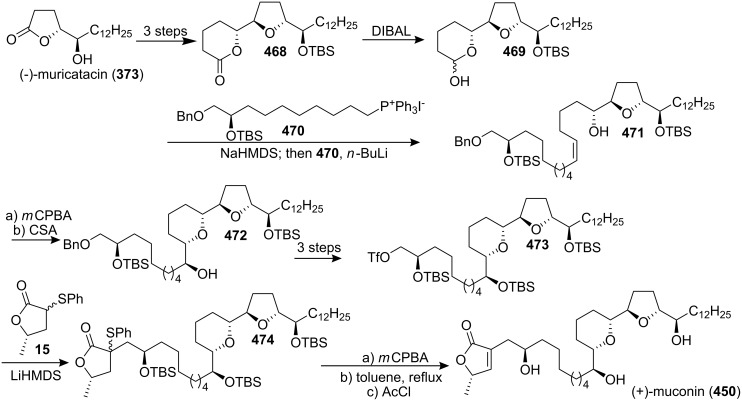
Total synthesis of muconin by the group of Yoshimitsu and Nagaoka.

In 2004, the group of Yoshimitsu and Nagaoka reported the total synthesis of (+)-muconin (**450**) starting from (−)-muricatacin (**373**) (Scheme 67) [143]. (−)-Muricatacin (**373**) was converted to δ-lactone **468**. Reduction of **468** with diisobutylaluminum hydride provided lactol **469**, the sodium alkoxide derivative of which subsequently underwent Wittig olefination with phosphonium compound **470** to give olefin **471**. **471** was oxidized with *m*CPBA to provide an epoxide whose opening with CSA gave tetrahydropyran **472**. The triflate **473** was reacted with the lithium enolate generated from the known α-thiophenyl γ-lactone **15** to provide lactone **474**; subsequent elimination and deprotection finished the total synthesis of (+)-muconin (**450**), whose spectroscopic and analytical data were consistent with those of the natural product.

### 6 Nonadjacent THF-THP rings

#### Total synthesis of mucocin

**Scheme 68:**
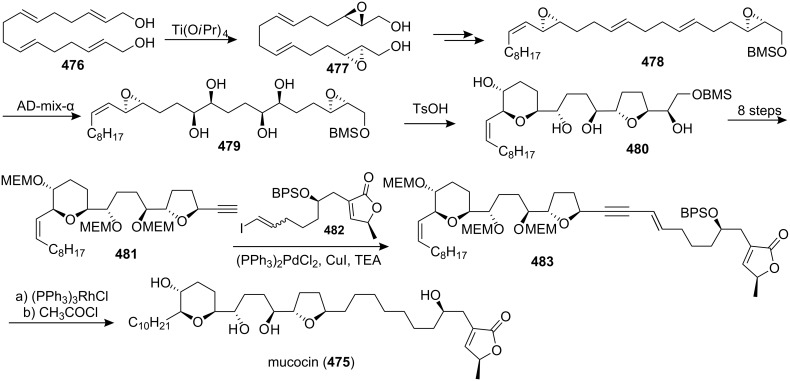
Total synthesis of mucocin by the group of Sinha and Keinan.

Mucocin (**475**), which was isolated from the leaves of *Rollinia mucosa*, was the first ACG reported that bears a hydroxylated THP ring along with a THF ring [144]. Mucocin was found to be quite active in the BST assay (IC_50_ 1.3 µg/mL) and showed selective inhibitory effect against A-549 (ED_50_ = 1.0 × 10^−6^ µg/mL) and PACA-2 (ED_50_ = 4.7 × 10^−7^ µg/mL) in a panel of six human solid tumor cell lines [144]. Its selective potency was up to 10,000 times that of adriamycin. Interestingly, mucocin was found to be as active as bullatacin in inhibition of oxygen uptake by rat liver mitochondria (LC_50_ 18 and 9 nM/mg protein, respectively). In 1998, the group of Sinha and Keinan reported the first total synthesis of mucocin *via* the “naked” carbon skeleton strategy (Scheme 68) [145]. All eight asymmetric centers in the key fragment **479** of the molecule were introduced by double AE reaction of **476** followed by double AD reaction of **478**. Treatment of **479** with a catalytic amount of TsOH induced the double ring closure to afford the nonadjacent THP-THF ring system **480**, which was then transformed into the alkyne **481**. Cross-coupling with Pd(PPh_3_)_2_Cl_2_ catalyst of **481** and **482** afforded enyne **483**. Homogeneous catalytic hydrogenation and acid-catalyzed deprotection of all four protecting groups in **483** afforded **475**, which was found to be identical (MS, ^1^H and ^13^C NMR, [α]_D_) with the naturally occurring mucocin.

**Scheme 69:**
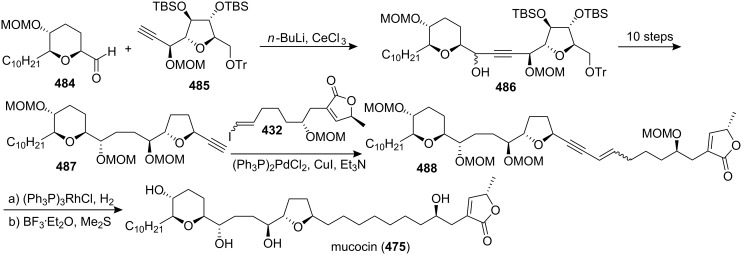
Total synthesis of mucocin by Takahashi’s group.

In 1999, Takahashi’s group also reported the total synthesis of mucocin (**475**) (Scheme 69) [146]. Reaction of aldehyde **484** with the lithiated alkyne **485** produced the alcohol **486** [147]. Then the complete carbon skeleton of **475** was assembled by joining **487** and **432** under Hoye’s conditions to give the labile enyne **488**, which underwent regioselective reduction followed by deprotection thus completing the total synthesis of **475**, whose spectral properties were indistinguishable from those of the natural product [144]. In 2002, the full procedure for this total synthesis was reported [148].

**Scheme 70:**
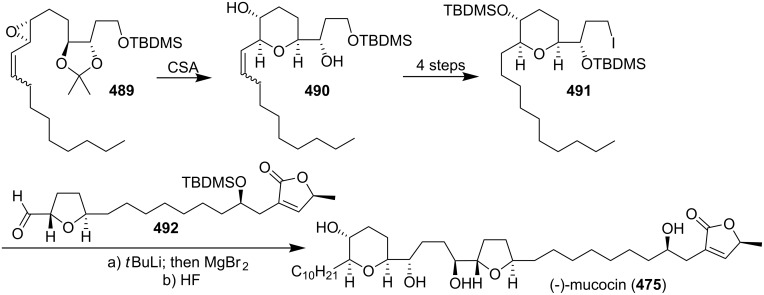
Total synthesis of (−)-mucocin by Koert’s group.

In 1999, Koert’s group reported the total synthesis of (−)-mucocin (**475**) by using a stereocontrolled coupling reaction (Scheme 70) [65]. An acid-catalyzed intramolecular 6-*endo* attack on the alkenyl epoxide of the acetonide in **489** afforded the THP ring **490**, which was transformed into the iodide **491** in 4 steps. Finally, addition of an organometallic reagent derived from **491** to the THF aldehyde **492** followed by deprotection provided (−)-mucocin (**475**) ([α]_D_ = –12.7, *c* 0.27 in CH_2_Cl_2_), which was found to be identical to the naturally occurring product in respect to the spectroscopical data. In 2000, the full details of this total synthesis were reported [149].

**Scheme 71:**
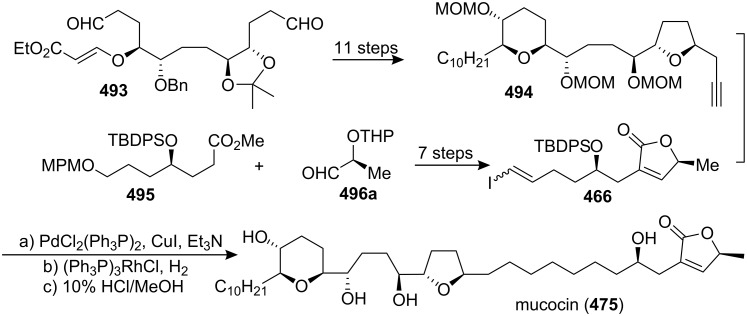
Total synthesis of mucocin by the group of Takahashi and Nakata.

In 2002, the group of Takahashi and Nakata reported the total synthesis of **475** based on the SmI_2_-induced reductive cyclization as a key step (Scheme 71) [150]. The THP ring in the central core **494** was constructed from **493** by the SmI_2_-induced reductive cyclization, whereas the *trans*-THF ring was synthesized by oxidative cyclization of a homoallylic alcohol. The γ-lactone **466** was synthesized by aldol condensation of chiral ester **495** and aldehyde **496a**. Finally, a Pd-catalyzed cross-coupling reaction of the THP/THF segment **494** and vinyl iodide **466** followed by hydrogenation and global deprotection finished the total synthesis of **475**.

**Scheme 72:**
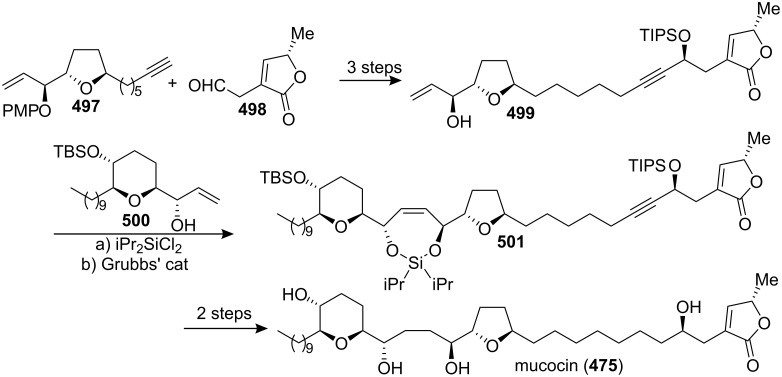
Total synthesis of mucocin by Evans’s group.

In 2003, Evans’s group reported the total synthesis of (−)-mucocin (**475**) by using a temporary silicon-tethered (TST) RCM homo-coupling reaction (Scheme 72) [151]. The enantioselective addition of the alkynyl zinc reagent derived from **497** to the aldehyde **498** furnished the propargylic alcohol. Protection of the alcohol as the triisopropylsilyl ether followed by deprotection of the *p*-methoxyphenyl ether afforded the allylic alcohol **499**. The TST-RCM cross-coupling reaction between **499** and **500** furnished **501** and completed the construction of the carbon skeleton of mucocin (**475**).

**Scheme 73:**
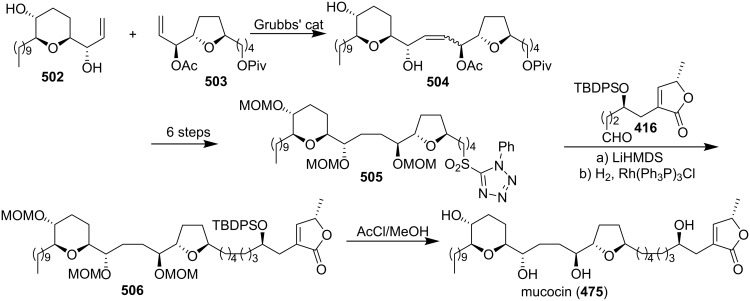
Total synthesis of mucocin by Mootoo’s group.

In 2005, Mootoo’s group reported the total synthesis of mucocin (**475**) in a three component modular approach based on olefinic coupling reactions (Scheme 73) [152]. They used a cross-metathesis on tetrahydropyran **502** and THF **503** to assemble a stereochemically complex bicyclic ether **504**, which was further elaborated to sulfone **505**. Then **505** was reacted with butenolide aldehyde component **416** in a Julia–Kocienski olefination to provide the mucocin framework **506**, which was converted to the natural product **475** after alcohol deprotection.

**Scheme 74:**
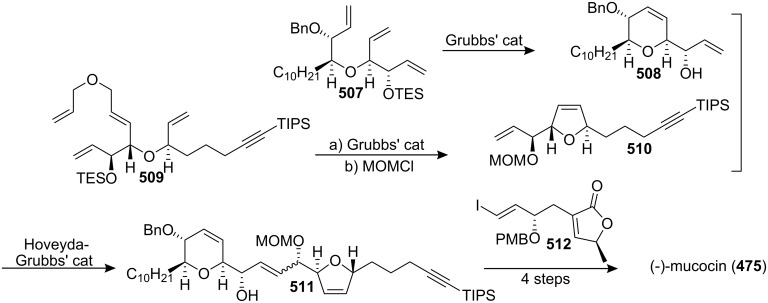
Total synthesis of (−)-mucocin by Crimmins’s group.

In 2006, Crimmins’s group reported the enantioselective total synthesis of (−)-mucocin (**475**) (Scheme 74) [153]. Both fragments **508** and **510** were prepared via an asymmetric glycolate aldol-RCM sequence. Then **508** and **510** were coupled through a cross-metathesis reaction to afford bicyclic ether **511**. The coupling of advanced acetylene **511** and known butenolide **512** finished the total synthesis of (−)-mucocin (**475**).

### 7 mono-THP ACGs

#### Total synthesis of Pyranicin

**Scheme 75:**
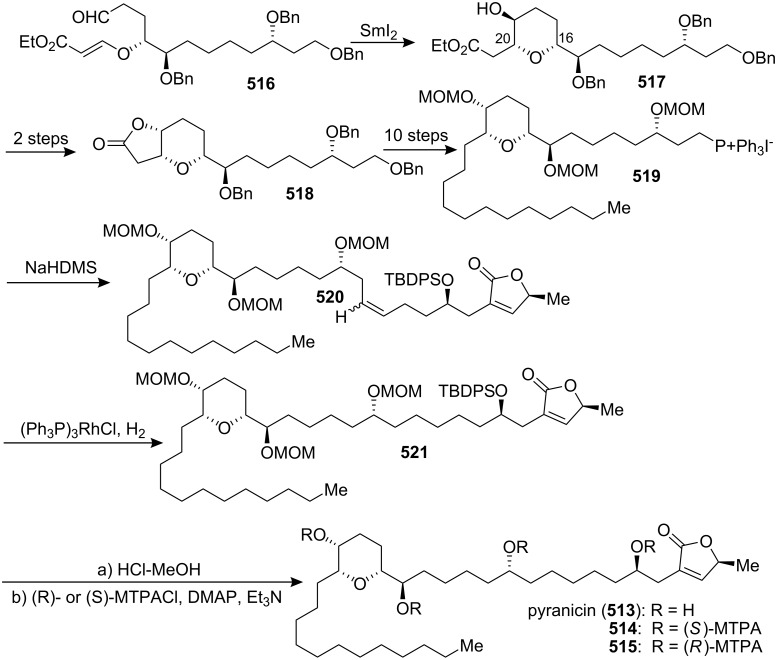
Total synthesis of pyranicin by the group of Takahashi and Nakata.

Pyranicin (**513**), which was isolated from the stem bark of *Goniothalamus giganteus*, was the first mono-THP acetogenin isolated [154]. The acetogenin **513** was quite active in the BST assay (LC_50_ = 0.3 µg/mL) [155] and showed selective inhibitory effects against PACA-2 cell lines (ED_50_ = 1.3 × 10^−3^ µg/mL) with potency 10 times that of adriamycin (ED_50_ = 1.6 × 10^−2^ µg/mL) [154]. In 2003, the group of Takahashi and Nakata reported the first total synthesis of **513** in a stereocontrolled manner (Scheme 75) [156]. SmI_2_-induced reductive cyclization of **516** afforded a 16,20-*cis*-19,20-*anti*-THP derivative **517**. Through utilization of Mitsunobu lactonization, stereoinversion at the C-19 position was achieved affording **518**, which was transformed into the phosphonium salt **519** through DIBAL reduction and Wittig reaction. Construction of the complete carbon skeleton of **513** was achieved through a Wittig reaction, then global deprotection of **521** produced pyranicin (**513**). The synthetic **513** showed [α]_D_^24^ +19.5 (*c* 0.55, CHCl_3_), while the [α]_D_^23^ value of natural **513** was reported to be –9.7 (*c* 0.008, CHCl_3_). However the NMR data of the corresponding MTPA esters (**514** and **515**) were revealed to be well matched with those reported. As the optical rotation of the natural product was measured at very low concentration, the difference might be due to experimental error or the presence of impurities.

**Scheme 76:**
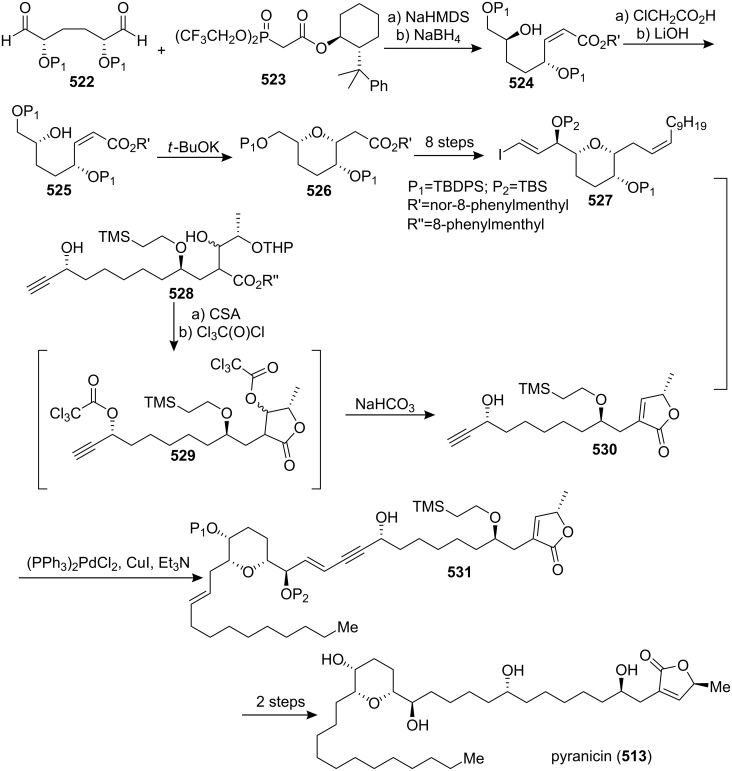
Total synthesis of pyranicin by Rein’s group.

In 2005, Rein’s group reported a convergent total synthesis of **513** employing asymmetric Horner-Wadsworth-Emmons (HWE) reactions (Scheme 76) [157]. Their synthesis began with the desymmetrization of *meso*-dialdehyde **522** through an asymmetric HWE olefination which gave the secondary alcohol **524**. A Mitsunobu reaction followed by basic hydrolysis of the resulting chloroacetate then gave the inverted product **525**. In the subsequent hetero-Michael cyclization, the desired *cis*-*cis*-THP **526** was formed, which was then transformed into the desired vinyl iodide **527**. Lactonization of **528** under acidic conditions gave the desired lactone **529** as a diastereomeric mixture, which was then treated with base to afford the propargylic alcohol **530**. The complete pyranicin framework was assembled through a Sonogashira coupling of **527** and **530**, giving ene-yne **531**. Finally, a selective diimide reduction followed by global deprotection using HF in MeCN afforded pyranicin (**513**). In 2006, they reported the full details of this total synthesis [158].

#### Total synthesis of Pyragonicin

**Scheme 77:**
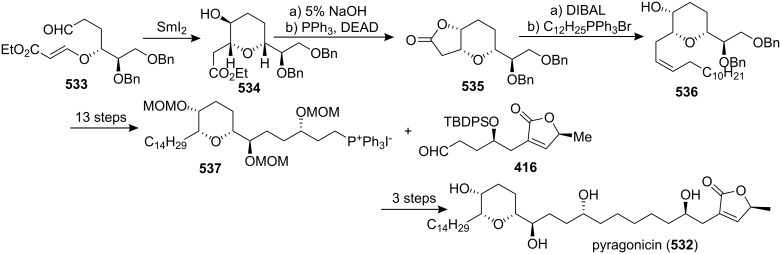
Total synthesis of proposed pyragonicin by the group of Takahashi and Nakata.

Pyragonicin (**532**), which was isolated from the stem bark of *Goniothalamus giganteus* [154], was active in the BST assay (LC_50_ = 0.9 µg/mL) [155] and showed a selective inhibitory effect against PACA-2 (ED_50_ = 5.8 × 10^−2^ µg/mL) [154]. In 2005, the group of Takahashi and Nakata reported the first total synthesis of the proposed structure of pyragonicin **532** (Scheme 77) [159]. SmI_2_-induced reductive cyclization of **533** gave THP ester **534**. Stereoinversion at the C-17 position was achieved using a Mitsunobu lactonization of **534**, subsequent DIBAL reduction and Wittig reaction afforded olefin **536**, which was transformed into the phosphonium salt **537**. Then Wittig reaction of aldehyde **416** completed the construction of pyragonicin (**532**). Compound **532** had spectroscopic data consistent with that of natural pyragonicin, but a different optical rotation.

**Scheme 78:**
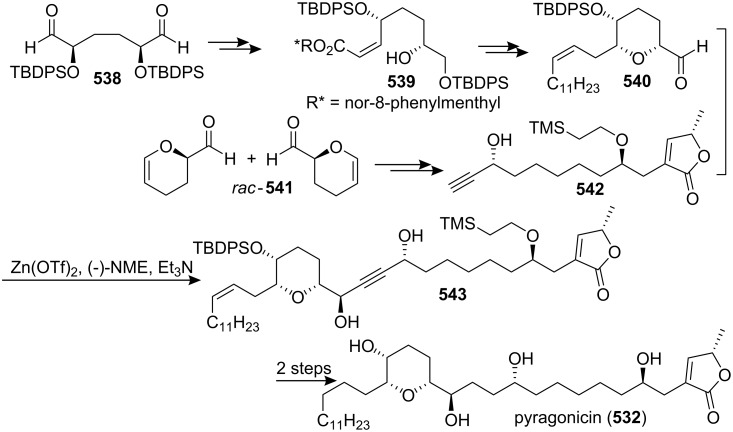
Total synthesis of pyragonicin by Rein’s group.

In 2005, Rein’s group also reported the total synthesis of pyragonicin (**532**) using the asymmetric Horner-Wadsworth-Emmons (HWE) methodology (Scheme 78) [160]. The THP-fragment **540**, which in turn was accessed from *meso*-dialdehyde **538** *via* an asymmetric HWE desymmetrization, coupled with **542**, which was also constructed from *rac*-**541** by a parallel kinetic HWE resolution, completed the total synthesis of pyragonicin (**532**). The spectroscopic data of **532** (IR, ^1^H and ^13^C NMR) were, within the normal error limits for such data, identical to those reported by McLaughlin. However, there was a strong discrepancy in the optical rotation. In 2006, the full details of this total synthesis were reported [158].

**Scheme 79:**
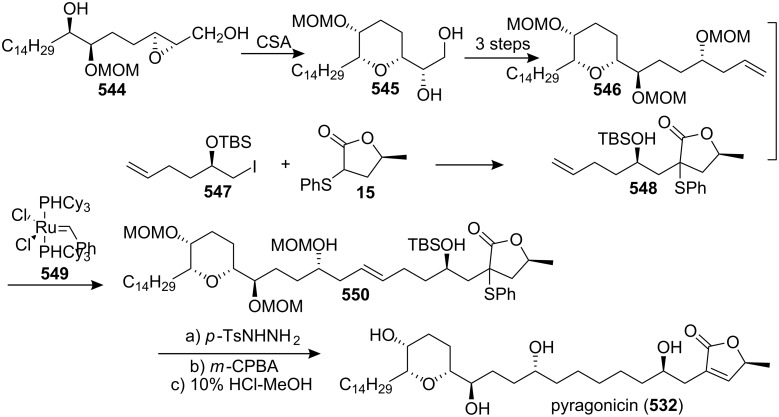
Total synthesis of pyragonicin by Takahashi’s group.

In 2006, Takahashi’s group described a second-generation synthesis of pyragonicin (**532**) (Scheme 79) [161]. The key step involved an olefin cross-metathesis between the THP segment **546** and the terminal γ-lactone residue **548** in the presence of Grubbs’ first-generation catalyst **549** affording the desired coupling product **550** exclusively. The olefin **550** underwent hydrogenation followed by *syn*-elimination of the sulfoxide and global deprotection to finish the total synthesis of pyragonicin (**532**).

### 8 Only γ-lactone ACGs

#### Total synthesis of squamostanal A

**Scheme 80:**
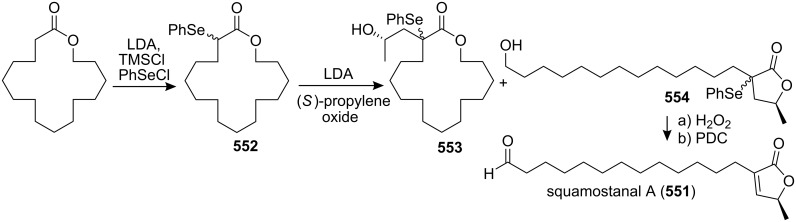
Total synthesis of squamostanal A by Figadère’s group.

Squamostanal A (**551**) was isolated from the seeds of *Annona squamosa* and characterized by usual spectroscopic methods (NMR, mass spectrometry, and circular dichroism) as (5*S*)-3-(12-formyldodecyl)-5-methyl-2,5-dihydrofuran-2-one [162]. In 1996, Figadère’s group reported the total synthesis of squamostanal A (**551**) in only 4 steps (Scheme 80) [163]. **552** was enolized and added to (*S*)-propylene oxide to afford the macrolactone **553** and butyrolactone **554**. After separation, **554** was first oxidized with H_2_O_2_ to afford the corresponding butenolide, and then treated with PDC to afford the desired product **551**, whose spectroscopic data were in accord with those of natural squamostanal A.

#### Total synthesis of diepomuricanin

**Scheme 81:**
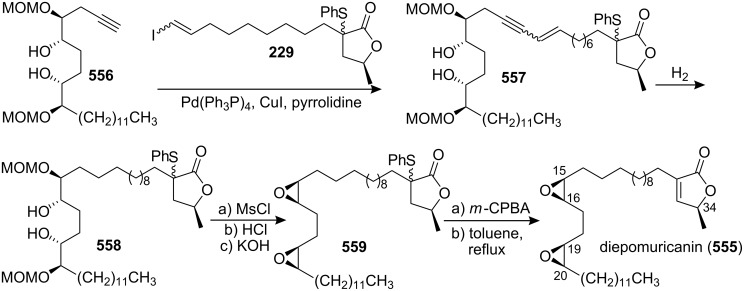
Total synthesis of diepomuricanin by Tanaka’s group.

Diepomuricanin (**555**), which was isolated from *Annona muricata* by Cavé’s group [164], was assumed to be a biosynthetic intermediate for tetrahydrofuranic annonaceous acetogenins. In 1996, Tanaka’s group reported the total synthesis of (15*S*,16*R*,19*S*,20*R*,34*S*)-diepomuricanin (**555**) (Scheme 81) [165]. A Pd-mediated cross-coupling reaction between **556** and **229** yielded enyne **557**, then catalytic hydrogenation followed by treatment with MsCl/Et_3_N, dil. HCl/MeOH and KOH/THF afforded **558**. Oxidation to the sulfoxide with *m*-CPBA/NaHCO_3_ and subsequent thermal elimination by refluxing in toluene led to (15*S*,16*R*,19*S*,20*R*,34*S*)-diepomuricanin (**555**). By comparing the IR, ^1^H and ^13^C NMR data and the optical rotation values (synthetic [α]_D_ +17.0; natural [α]_D_ +13.5), the absolute configuration of diepomuricanin was likely to be 15*S*,16*R*,19*S*,20*R*,34*S*.

#### Total synthesis of muricatacin

**Scheme 82:**
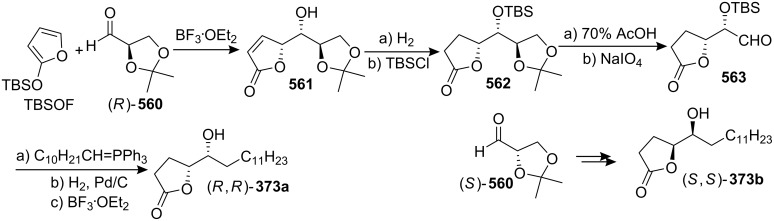
Total synthesis of (−)-muricatacin [(*R*,*R*)-**373a**] and its enantiomer (+)-muricatacin [(*S*,*S*)-**373b**] by the group of Rassu and Casiraghi.

Muricatacin, an acetogenin derivative that showed cytotoxic activity against certain human tumor cell lines, had been isolated from the seeds of *Annona muricata* [166], and remarkably, the natural compound comprises both (−)-muricatacin [(*R*,*R*)-**373a**] and its enantiomer (+)-muricatacin [(*S*,*S*)-**373b**]. In 1997, the group of Rassu and Casiraghi reported the synthesis of both enantiomers of muricatacin, (*R*,*R*)-**373a** and (*S*,*S*)-**373b** (Scheme 82) [167]. (+)-(*R*)-glyceraldehyde acetonide (*R*-**560**) coupled with (*tert*-butyldimethylsilyl)-2-hydroxyfuran (TBSOF) afforded the 4,5-*syn*-configured adduct **560**. Compound **561** was subjected to sequential catalytic hydrogenation and protection of the OH function afforded the seven-carbon intermediate **562**. The oxidative removal of the C-7 carbon atom generated the six-carbon aldehyde **563**. Wittig olefination of aldehyde **563** with the appropriate C_11_ ylide followed by catalytic hydrogenation and BF_3_ etherate-promoted desilylation afforded (−)-muricatacin [(*R*,*R*)-**373a**]. Its enantiomer (+)-muricatacin [(*S*,*S*)-**373b**] was synthesized from (+)-(*S*)-glyceraldehyde acetonide (*S*-**560**) using the same procedure.

**Scheme 83:**
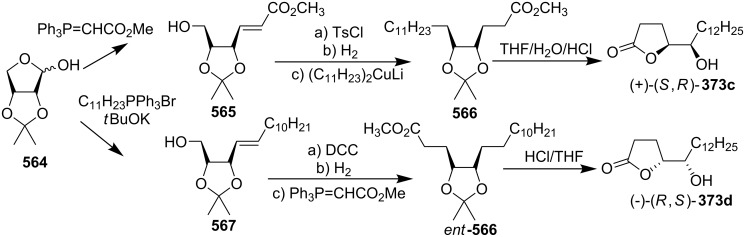
Total synthesis of *epi*-muricatacin (+)-(*S*,*R*)-**373c** and (−)-(*R*,*S*)-**373d** by Scharf’s group.

In 1997, Scharf’s group reported the total synthesis of both enantiomers of *epi*-muricatacin (+)-(*S*,*R*)-**373c** and (−)-(*R*,*S*)-**373d** by means of a change in the sequence of side-chain introduction from the same chiral precursor **564** (Scheme 83) [168].

**Scheme 84:**
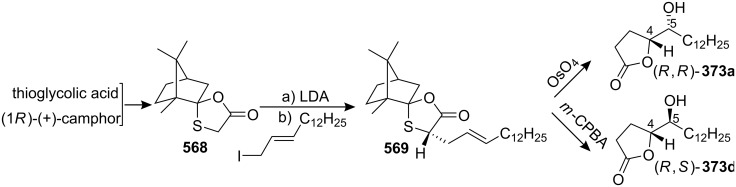
Total synthesis of (−)-muricatacin **373a** and 5-*epi*-(−)-muricatacin **373d** by Uang’s group.

In 1998, Uang’s group reported the synthesis of (−)-muricatacin [(*R*,*R*)-**373a**] and 5-*epi*-(−)-muricatacin [(*R*,*S*)-**373d**] from thioglycolic acid employing (*1R*)-(+)-camphor as the chiral auxiliary (Scheme 84) [169]. Oxidation of **569** with OsO_4_ afforded **373a**, while *m*-CPBA promoted oxidation of **569** afforded **373d**.

**Scheme 85:**
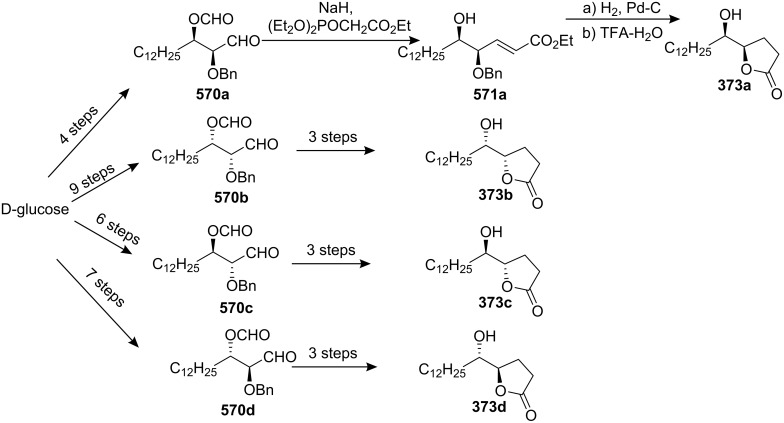
Total synthesis of four stereoisomers of muricatacin by Yoon’s group.

In 1998, Yoon’s group reported the synthesis of four stereoisomers of muricatacin **373a**–**d** through the reaction of corresponding aldehydes **570a**–**d** [170], prepared from D-glucose, with the anion of triethylphosphonoacetate followed by reduction and cyclization under acidic conditions (Scheme 85).

**Scheme 86:**
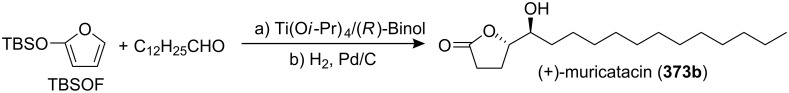
Total synthesis of (+)-muricatacin by Figadère’s group.

In 1998, Figadère’s group reported the synthesis of muricatacin (**373b**) through addition of TBSOF to an achiral aldehyde promoted by Ti(O*i*Pr)_4_ in the presence of (*R*)-1,1′-bi-2-naphthol (BINOL) followed by hydrogenation (Scheme 86) [171]. It is worth noting that the major *threo* product was obtained with 90% ee through this titanium-mediated addition of TBSOF to tridecanal in (*R*)-BINOL at −20 °C in Et_2_O.

**Scheme 87:**
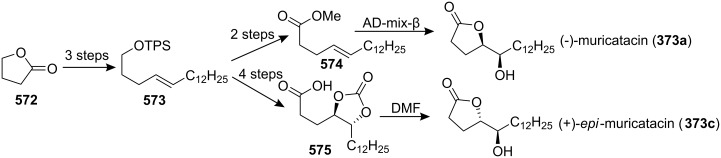
Total synthesis of (+)-*epi*-muricatacin and (−)-muricatacin by Couladouros’s group.

In 1999, Couladouros’s group reported the total synthesis of (−)-muricatacin (**373a**) and (+)-*epi*-muricatacin (**373c**) from the same γ-lactone **572** (Scheme 87) [172].

**Scheme 88:**
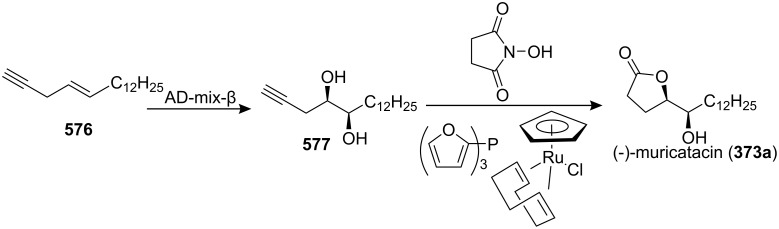
Total synthesis of muricatacin by Trost’s group.

In 1999, Trost’s group reported the total synthesis of muricatacin (**373a**) through ruthenium-catalyzed cycloisomerization-oxidation on **577**, which was synthesized from enyne **576** *via* asymmetric dihydroxylation (Scheme 88) [173].

**Scheme 89:**
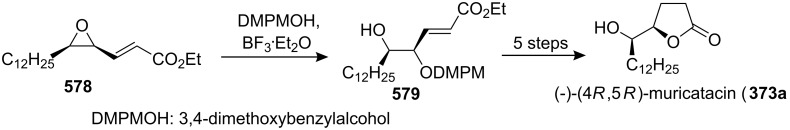
Total synthesis of (−)-(4*R*,5*R*)-muricatacin by Heck and Mioskowski’s group.

In 2000, the group of Heck and Mioskowski reported the total synthesis of (−)-(4*R*,5*R*)-muricatacin (**373a**) using as a key step a regio- and stereospecific ring-opening of a substituted vinyl epoxide **578** under Lewis acid catalysis (Scheme 89) [174].

**Scheme 90:**
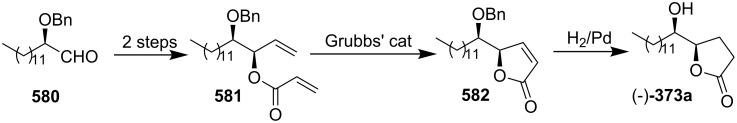
Total synthesis of muricatacin (−)-**373a** by the group of Carda and Marco.

In 2002, the group of Carda and Marco reported the stereoselective synthesis of muricatacin (−)-**373a** through a ring-closing metathesis (Scheme 90) [175]. The acrylate **581**, which was synthesized from (*R*)-2-benzyloxytetradecanal **580**, underwent the RCM reaction, thus furnishing lactone **582**. Hydrogenation of **582** finished the total synthesis of muricatacin (−)-**373a**.

**Scheme 91:**
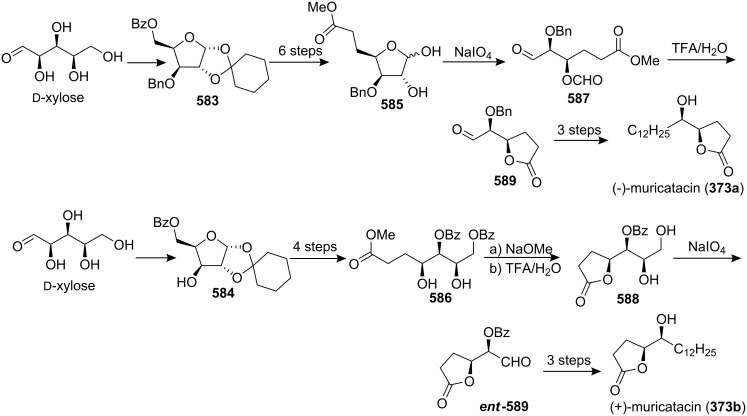
Total synthesis of (−)- and (+)-muricatacin by Popsavin’s group.

In 2003, Popsavin’s group reported a novel general approach using an enantiodivergent synthesis of **373a** and **373b** from D-xylose (Scheme 91) [176]. The lactol **585**, which was obtained from **583**, was transformed into the formate **587** through oxidation. **587** was treated with aqueous trifluoroacetic acid to yield the *α*-lactone **589**. 5-*O*-Benzoyl-1,2-*O*-cyclohexylidene-α-D-xylofuranose **584** was transformed into the corresponding saturated ester **586** through a Wittig olefination followed by catalytic hydrogenation, and **586** was treated with sodium methoxide in methanol to furnish the hydroxylactone **588**, whose oxidation afforded aldehydo-lactone *ent*-**589**. The chiral synthons **589** and *ent*-**589** were converted into the targets **373a** and **373b** through a known two-step sequence [177].

**Scheme 92:**
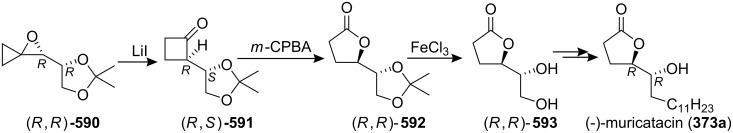
Total synthesis of (−)-muricatacin by the group of Bernard and Piras.

In 2003, the group of Bernard and Piras reported the total synthesis of (−)-(4*R,5R*)-muricatacin (**373a**) from cyclobutanone **591**, which was obtained by lithium salt catalyzed ring expansion of the optically pure oxaspiropentane **590***. (R*,*S*)-**591** was transformed into the corresponding γ-lactone (*R*,*R*)-**592** through a Baeyer-Villiger oxidation. Synthesis of the γ-lactone (*R*,*R*)-**593** constituted a formal synthesis of (−)-muricatacin (**373a**) (Scheme 92) [178].

**Scheme 93:**
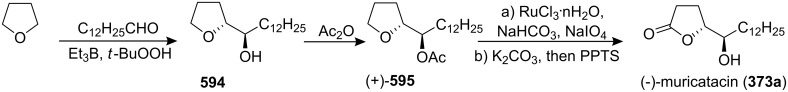
Total synthesis of (−)-muricatacin by the group of Yoshimitsu and Nagaoka.

In 2003, the group of Yoshimitsu and Nagaoka reported the total synthesis of (−)-muricatacin (**373a**) (Scheme 93) [179] through α-C-H hydroxyalkylation of THF with tridecanal using triethylborane-TBHP, which provided alcohols **594**. Then α′-C-H oxidation of THF (+)-**595** with ruthenium tetroxide under modified Sharpless conditions followed by deprotection finished the total synthesis of (−)-muricatacin (**373a**). This study presented a novel method for C-H bond functionalization as a means for preparing γ-(hydroxyalkyl)-γ-butyrolactones.

**Scheme 94:**
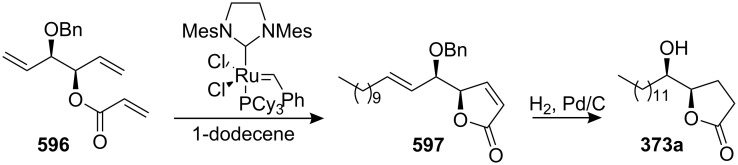
Total synthesis of (−)-muricatacin by Quinn’s group.

In 2004, Quinn’s group reported the total synthesis of (−)-muricatacin (**373a**) (Scheme 94) [180] by using the highly regioselective and stereoselective tandem ring-closing/cross metathesis reaction of **596** to construct the lactone and the alkyl chain in **597**. Then (−)-muricatacin (**373a**) was obtained by catalytic hydrogenation/hydrogenolysis of **597**.

#### Total synthesis of montecristin

**Scheme 95:**
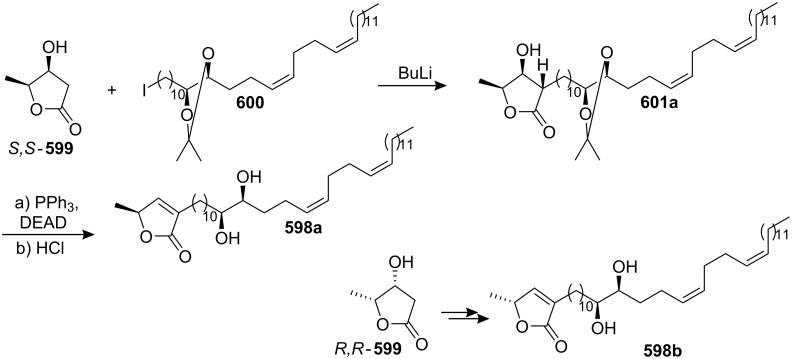
Total synthesis of montecristin by Brückner’s group.

(+)-Montecristin (**598**) isolated in 1997 from the roots of *Annona muricata* [181] might be an intermediate between the less and the more oxygenated acetogenins. In 2001, Brückner’s group reported the total synthesis of both **598a** and **598b** (Scheme 95) [182], and by comparing their specific rotations with those of montecristin, demonstrated that **598a** was *ent*-5-*epi*-montecristin while **598b** was the enantiomer of (+)-montecristin. Alkylating the dilithiated hydroxylactone *S*,*S*-**599** with iodide **600** delivered *trans*-alkylated hydroxylactone **601a**. The ensuing β-elimination of **601a** followed by acetonide cleavage finished the total synthesis of **598a**, while **598b** was prepared from *R*,*R*-**599** using the same procedure.

#### Total synthesis of acaterin

**Scheme 96:**
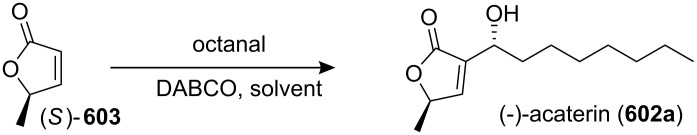
Total synthesis of (−)-acaterin by the group of Franck and Figadère.

Acaterin (**602a**), which was isolated from a culture broth of *Pseudomonas* sp. A92 by Endo’s group in 1992 [183], is an inhibitor of acyl-CoA:cholesterol acyltransferase (ACAT) [183]. In 2002, the group of Franck and Figadère reported the synthesis of (−)-acaterin (**602a**) through the first application of the Baylis–Hillman reaction to α,β-unsaturated lactone (*S*)-**603** (Scheme 96) [184].

**Scheme 97:**
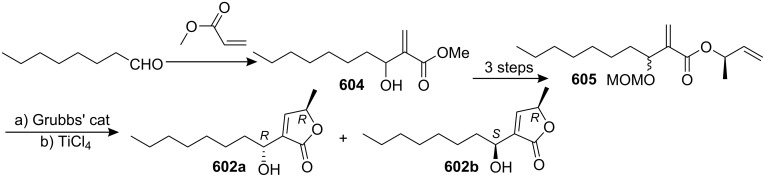
Total synthesis of (−)-acaterin by Singh’s group.

In 2002, Singh’s group reported a short and efficient synthesis of acaterin from **604** (Scheme 97) [185], which was constructed from caprylic aldehyde and methyl acrylate through a Baylis–Hillman reaction. Ring closing metathesis reaction on **605** using Grubbs’ catalyst followed by deprotection afforded natural (−)-acaterin (**602a**) and its diastereomer (**602b**).

**Scheme 98:**
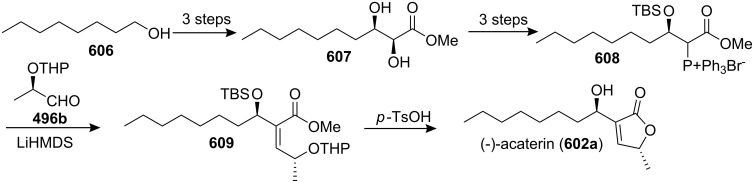
Total synthesis of (−)-acaterin by Kumar’s group.

In 2003, Kumar’s group reported the total synthesis of (−)-acaterin (**602a**) (Scheme 98) [186]. Starting from octan-1-ol, the phosphonium salt **608** was obtained by employing the Sharpless AD procedure and a Wittig olefination. Then the coupling of phosphonium salt **608** with aldehyde **496b** and subsequent cyclization afforded **602a**.

#### Total synthesis of rollicosin

**Scheme 99:**
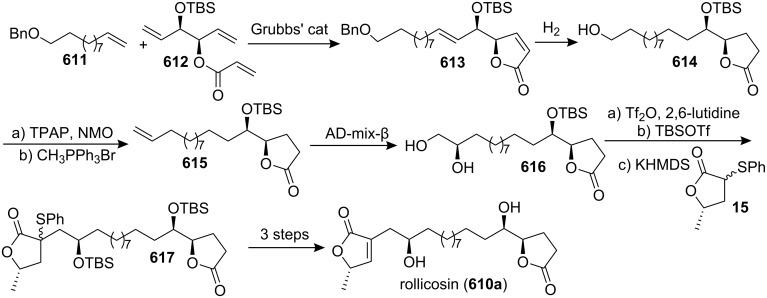
Total synthesis of rollicosin by Quinn’s group.

Rollicosin (**610a**), isolated in low yield from *Rollinia mucosa* in 2003, was a new subclass of acetogenins containing two terminal γ-lactones [187]. Quinn’s group reported the first total synthesis of rollicosin in 2005 using a tandem RCM/CM strategy for allyl butenolide preparation (Scheme 99) [188]. Butenolide **613** was produced by tandem RCM/CM with initial RCM of acrylate **612** preceding CM with the benzyl ether of 10-undecen-1-ol (**611**). **613** was exposed to H_2_ in the presence of Pd/C to effect removal of the benzyl ether and concomitant alkene reduction to provide alcohol **614**, TPAP oxidation to the corresponding aldehyde and one-carbon Wittig homologation then gave terminal alkene **615**. Treatment of **615** with AD-mix-β provided diol **616**, which after suitable protection was coupled with the enolate of **15** to produce **617**. Oxidation of sulfide **617** and thermal elimination followed by TBS deprotection provided rollicosin (**610a**), which displayed spectral data (IR, ^1^H and ^13^C NMR) and optical rotation consistent with that of naturally occurring rollicosin.

**Scheme 100:**
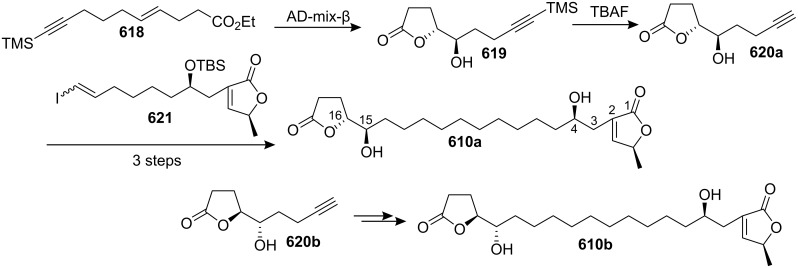
Total synthesis of Rollicosin by Makabe’s group.

In 2005, Makabe’s group reported the total synthesis of (4*R*,15*R*,16*R*,21*S*)-rollicosin (**610a**) and (4*R*,15*S*,16*S*,21*S*)-rollicosin (**610b**) (Scheme 100) [189]. Sharpless AD using AD-mix-β on **618** furnished lactone **619**. The hydroxy lactone **620a** and the α,β-unsaturated lactone **621** were coupled by the Sonogashira cross-coupling reaction. Subsequent diimide reduction and deprotection afforded **610a**. (4*R*,15*S*,16*S*,21*S*)-Rollicosin (**610b**) was also obtained starting from **620b** using the same procedure as that employed for **610a**. In 2006, the full details of this total synthesis were reported [190].

#### Total synthesis of squamostolide

**Scheme 101:**
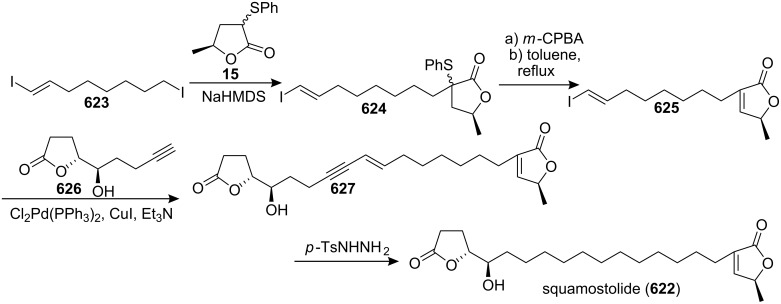
Total synthesis of squamostolide by Makabe’s group.

Squamostolide (**622**), which was isolated from *Annona squamosa* by Wei’s group [191], showed a remarkably weak inhibitory activity compared to ordinary acetogenins such as bullatacin [191]. In 2006, Makabe’s group reported the total synthesis of squamostolide (Scheme 101) [190]. The lactone **622** was obtained by alkylation of the enolate prepared from **15** using NaHMDS with diiodide **623**. The α,β-unsaturated lactone **625** was obtained after oxidation of **624** with *m*CPBA followed by thermal elimination of the resulting sulfoxide. Then segments **626** and **625** were coupled by a Sonogashira reaction to furnish product **627**. Diimide reduction with *p*-TsNHNH_2_ and NaOAc in ethylene glycol diethyl ether under reflux afforded squamostolide (**622**). The optical rotation, melting point, ^1^H NMR, and ^13^C NMR spectra of the synthetic **622** were in good agreement with those of the reported values.

#### Total synthesis of tonkinelin

**Scheme 102:**
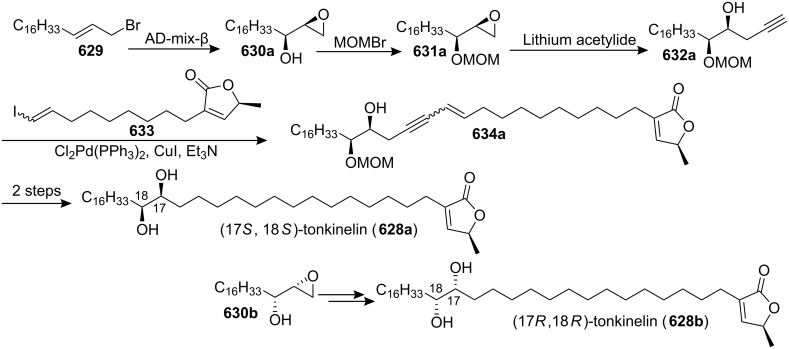
Total synthesis of tonkinelin by Makabe’s group.

Tonkinelin (**628a**), which has a simple structure in the acetogenins (compared with other types of ACGs posessing THF ring or THP ring), was isolated from *Uvaria tonkinesis* in 1996 by Chen’s group [192]. This compound has two hydroxyl groups at C-17 and C-18 position, and possesses an α,β-unsaturated γ-lactone which can be seen in ordinary ACGs. In 2007, Makabe’s group reported the total synthesis of tonkinelin **628a** (Scheme 102) [193]. Asymmetric dihydroxylation of **629** by the Sharpless procedure using AD-mix-α and spontaneous epoxide formation afforded epoxy alcohol **630a**. Then the hydroxyl group of **630a** was protected as a methoxymethyl ether (MOM ether) to give compound **631a**. Alkynylation of **631a** afforded **632a**, and Sonogashira cross-coupling reaction of **632a** with **633** gave enyne **634a**. Diimide reduction of **634a** followed by deprotection of the MOM ether with BF_3_·Et_2_O afforded **628a**. The other candidate **628b** was synthesized from **630b** using the same procedure. By comparison of the optical rotation of the synthetic candidates and the natural compound, they suggested that the absolute configuration of natural tonkinelin was likely to be (17*S*,18*S*).

## Conclusion

Annonaceous acetogenins are a relatively new class of bioactive naturally occurring products. The difficulty of isolating these compounds and elucidating their structures makes them a challenging target for total synthesis. Their wide spectrum of biological properties is probably the most intriguing and exciting domain, and the future will show whether it is possible to disclose the structure-activity relationship, probably on the basis of synthetic derivatives. Furthermore it will be useful to look for simplifications of the structure without loss of activity. As a result of these investigations, it will not be surprising if annonaceous acetogenins or related compounds with structural modifications might, in the near future, play a significant role in cancer therapy *via* an original mechanism of action. Hence we believe it is worthwhile to observe further developments in the field of annonaceous acetogenins.
